# Effect of pre-germination temperature conditions on germination characteristics of temperate grassland species

**DOI:** 10.1186/s12862-025-02424-5

**Published:** 2025-08-14

**Authors:** Réka Kiss, Katalin Lukács, Ágnes Tóth, Benedek Tóth, Kenz Raouf Samraoui, Rita Engel, Balázs Deák, Orsolya Valkó

**Affiliations:** 1 ’Lendület’ Seed Ecology Research Group, Institute of Ecology and Botany, HUN-REN Centre for Ecological Research, Vácrátót, Hungary; 2 National Laboratory for Health Security, HUN-REN Centre for Ecological Research, Budapest, Hungary; 3 Department of Ecology, University of Szeged, Szeged, Hungary; 4 MTA-SZTE ‘Momentum’ Applied Ecology Research Group, Department of Ecology, University of Szeged, Szeged, Hungary; 5 University of South Bohemia, Faculty of Science, České Budějovice, Czech Republic; 6 Institute of Botany of the Czech Academy of Sciences, Průhonice, Czech Republic

**Keywords:** Climate change, Germination capacity, Grassland specialist species, Seed dormancy, Stratification

## Abstract

Understanding germination characteristics, including optimal stratification requirements of target species, is necessary for supporting grassland restoration yet poorly understood. This knowledge is essential for effective conservation, particularly with climate change altering germination conditions and thus germination capacity of plants. Here we studied the effect of three different durations of warm dry and cold wet stratification treatments, and their combinations in a full factorial design (in total 15 different pre-germination treatments), on the germination capacity of 48 grassland species native to Central Europe. Stratification treatments modelled present and forecasted summer (1–3 months warm period) and winter (1–3 months cold period) temperature conditions, while the study of the combined effect of these treatments is especially important in spring-germinating species. As response variables, we calculated relative response indexes and germination uncertainties of each species separately and applied general linear models to study the effect of treatments on these variables. We found clear effect of warm- or cold stratification on relative response indexes only in four species: strong positive response to warm stratification was found in *Silene conica*, while strong positive response to cold stratification was found in *Agrimonia eupatoria*, *Echium vulgare*, and *Plantago lanceolata*. The responses to treatment combinations were contradictory or lacked clear trends in most of the species. Germination uncertainty in general was high for all species, supporting the fact that Central European grassland species often rely on bet hedging as risk spreading strategy, to avoid unfavourable conditions during seedling establishment.

**Supplementary Information:**

The online version contains supplementary material available at 10.1186/s12862-025-02424-5.

## Background

Grasslands are species-rich communities that are among the most diverse ecosystems in the world [1]. Considering their importance in providing various ecosystem services, such as regulation of water supplies, biomass production, and pollination; their conservation is of great importance [2–4]. Conservation of grasslands involves the protection of existing ones and the restoration of degraded and lost ones [3, 5]. Grassland degradation and loss is primarily driven by land-use changes, i.e. management intensification or abandonment, as well as infrastructural development [1]. These changes lead to considerable declines, with grassland loss exceeding 70% in certain regions of Europe [1]. As grassland loss and degradation have intensified, the demand for effective restoration strategies has increased to counter their adverse ecological consequences [6]. Global grassland restoration actions gained special momentum in the UN Decade on Ecosystem Restoration [3] and in Europe the EU Nature Restoration Law will also facilitate the restoration of several endangered grassland types. Grassland restoration often requires active interventions including species (re)introduction to the target areas [7]. Seed sowing is a widely used method for seed introduction [8]; seed mixtures composed of native plants of local provenance can be efficiently used in grassland restoration actions [9–11]. Although the amount of available seeds for restoration purposes is limited, the demand for restoration increases continuously [4, 12, 13]. Therefore, a solution for this problem might be the enhancement of the efficiency of seed sowing based restoration. This can be either achieved by seed enhancement techniques (i.e., seed priming and coating or applying pre-treatments that alleviate seed dormancy [14, 15], or by conscious seed use (i.e., sowing in adequate period for successful germination and establishment [14]. A deeper understanding of seed germination requirements, particularly seed dormancy (hereafter “dormancy”) breaking methods for the target species, could further improve seed use efficiency. However, although this information should be considered during restoration projects, the existing knowledge gaps delay their effective consideration.

Seed germination is induced by a set of environmental signals related to temperature, water availability, and light conditions [16]; upon meeting the adequate conditions seed germination occurs. Favourable conditions for germination and seed dispersal often occur in different periods of the year; plants overcome the unfavourable conditions of the intermediate periods by producing dormant seeds. Seed dormancy is the mechanism that prevents seed germination not only under unfavourable conditions but also under apparently favourable ones [17, 18]. The presence of a fraction of seeds that remains dormant under favourable conditions is a risk-spreading strategy used by plants; this strategy, known as bet-hedging [19]reduces year-to-year variation in fitness through prolonged seed dormancy and variation in germination timing [20–22]. Dormancy is present in 50–90% of the global wild flora [16]. The type of dormancy as well as the dormancy breaking mechanisms depend on various factors [23–25] such as phylogeny [26] geography [27, 28] climate [29–31] environmental predictability [23, 28] and habitat type [27, 32]. There are five major dormancy types and several subtypes; the most common dormancy types are physiological dormancy (PD, when the fully developed embryo possesses an inhibiting mechanism that prevents emergence), and physical dormancy (PY, when the seed possesses a hard seed coat impenetrable to water [16, 17, 32]). Less frequent types include morphological dormancy (MD, when the embryo must develop further before germination), morphophysiological dormancy (MPD, a combination of PD and MD) and combinational dormancy (CD, a combination of PD and PY) [16]. Dormancy breaking mechanisms vary between dormancy types: for some species a dry period following seed dispersal is enough to reduce and eliminate dormancy (dry after-ripening), while others require stratification (exposed to warm or cold temperature, which are signals of summer or winter periods), scarification (mechanical injury of the seed coat to allow water uptake) or the combination of different conditions and treatments. The optimal duration and intensity of these conditions are species-specific, and if not met, instead of germination seed become dormant (secondary dormancy) or loss viability [33, 34]. On the long run, this leads to changes in grasslands vegetation-structure and to regional extinctions of plant populations.

Despite their importance, dormancy type and dormancy breaking mechanisms are unknown for many species and many regions [35]. Besides, intraspecific differences between populations and even individuals exist, which further deepens the knowledge gap [36]. This knowledge gap not only decreases the efficiency of restoration based on seed sowing, but it also poses challenges in the face of climate change, even in regions where there is more data available on the dormancy types of plant species, such as in temperate Europe [16, 37, 38]. Climate change is predicted to raise temperatures in Central Europe: the warming will be the greatest in spring periods and lowest in autumns, while great variability in winter warming makes this season very unpredictable [39]. Beside warming, droughts will become more frequent and more severe especially during growing seasons [40, 41]. In Hungary the historical mean temperature ranged between 10°C-11°C, with −2  °C-1 °C in winter and 19°C-21°C in summer [42]. Mean temperatures are projected to increase by an average of 2.6°C by 2050; by 2.5°C in winters and by 3.7°C in summers [42]. In the past, the longer cold winters (~ 3 months) were coupled with shorter summers (~ 2 months); however, these conditions are expected to change, resulting in shorter cold periods and longer warm periods, with more temperature extremes in both seasons [39, 42, 43]. Without the basic understanding of the germination requirements, dormancy and germination characteristics, it is hard to forecast species- and community-level responses to changing environment (i.e. climate change [21, 44] and to improve the efficiency of seed-based restoration actions [14, 45, 46].

In our work, we conducted an experimental simulation to study the optimal temperature range and duration necessary to break dormancy and induce germination for target species. For this purpose, we selected 48 plant taxa typical of various grassland habitats in Central Europe. We explored the germination response of these species to three different durations of warm dry and cold wet stratification treatments, and their combinations in a full factorial design (in total 15 different pre-germination treatments). The treatments were designed to reflect current and forecasted seasonal conditions of the study region. Our questions were: (i) What is the effect of the longer warm periods and shorter cold periods on the germination success and dynamics of the studied species? (ii) What is the effect of combined warm and cold periods on the germination success and dynamics of the studied species? By addressing these questions, we aimed to contribute to filling the knowledge gap regarding the germination requirement of Central European plants. Including multiple species in our study, we enhanced our ability to form community-level predictions and design effective restoration plans in the future.

## Materials and methods

### Pre-germination treatments

We selected 48 typical grassland species for this experiment, that represent well the target species pool of restoration projects in lowland grasslands in Hungary (Table S1, nomenclature follows Király (2009) [47]. The seeds were purchased from a seed producer of native species who produced the seeds originating in the Kiskunság region (Central Hungary) in a propagation site located also in Kiskunság region. Following collection, seeds were cleaned by the producer and stored under dry conditions until the start of the experiment in the upcoming spring. A batch of 125 seeds of each species (25 seeds in five replicates) was subjected to each stratification treatment (in total 1,875 seeds per species). Stratification treatments were designed to model the changes in the length of the summer and winter periods: we used one-, two- or three months of warm dry stratification (warm stratification (W), at 25°C), one-, two- or three months of cold wet stratification (cold stratification (C), at 4°C) and the full factorial combination of the treatments (WC) (Table S2). Under natural conditions short-warm periods and longer cold periods were characteristic of the study region in the past; under climate change the length of warm periods increases while that of cold periods decreases [39, 42, 43]. The combination of treatments is important especially in case of spring-germinating species, which are affected both by the summer and winter conditions, so changes in both seasons will affect their germination success. Stratification treatments were selected to correspond both to seasonal projections (shortening winters with 1°C-4°C, longer summers with 23°C-25°C) [42] and to the literature defining temperatures adequate to exert effect on seeds [17].

For all pre-germination treatments seeds were placed between two one-layered filter papers in zip-lock bags. In cold stratification treatments filter papers were moistened with distilled water. Zip-lock bags containing the seeds of the 48 species were subjected to complete darkness by wrapping them in two layers of aluminium foil. Packs subjected to warm stratification were kept in a dark room, while bags subjected to cold stratification were kept in the refrigerator during the stratification period till the germination experiment.

### Germination experiment

At the end of the stratification periods, five replicates of 25 seeds per species were placed in five Petri dishes (110 mm in diameter) lined with one layer of filter paper (Whatman™, grade: 597, thickness: 180 μm), which was moistened with 3–4 ml of distilled water depending on the size of the seeds. Petri dishes were put in a growing chamber (type: PHCbi MLR-352, with 15 fluorescent tubes (Panasonic FL40SS ENW/37), each emitting white light of 53.4 µmol/m^2^/s) under 12 h photoperiod on 20/10°C (day/night). During day periods all fluorescent tubes were turned on, while at night they were all turned off. The germination experiment lasted for four weeks. Seedling emergence was verified weekly, germinated propagules were counted and removed from Petri dishes. Due to the limited capacity of the growing chambers, we needed to germinate seeds in four separate cycles (Table S2). In each of the four germination cycles, all the 48 species were germinated (please see the exact treatments per germination cycles in Table S2), along with five replicates of 25 untreated seeds (dry storage in room temperature, 19°C) of each species as controls (K).

### Statistical analysis

For the statistical analysis we calculated the Relative Response Index from the number of seedlings germinated (RRI) [48] and the germination uncertainty (U) [49–51] for each species in each treatment separately. RRI shows the effects of the treatments compared to the control and it was calculated as follows:

$${\text{RRI }}={\text{ }}({{\text{G}}_t}--{{\text{G}}_k})/\left( {{{\text{G}}_t}+{\text{ }}{{\text{G}}_k}} \right)$$

where G_t_ and G_k_ are the number of seedlings germinated in a particular treatment (G_t_) and in the untreated control (G_k_), respectively. RRI ranges between −1 and +1, zero means that the control and the treatment are not different. U measures the degree of germination dispersion, a lower value being associated with a more concentrated germination in time, while a higher number means a prolonged germination period. U was calculated using the *germinationmetrics* package [52]; to calculate and compare U, we omitted treatments where germination was not detected in given treatments or mean germination was below 4% (i.e., one seed germinated from 25 seeds in a Petri dish, see Table S4 and Table S5). We removed outliers from RRI data and added +2 to each RRI score and +1 to each U to be suitable to analyse it with Gamma distributed generalised linear models (GLMs); we used stratification treatments as explanatory variables (fix factor, 15 levels), RRI and U scores as dependent variables. GLMs with binomial distribution were used to compare mean germination success in the control treatments of the four germination cycles. All statistical analyses were performed in R statistical environment (version 4.3.2) [53]. Four species (*Galium boreale*, *G. verum*, *Salvia austriaca* and *Schoenus nigricans*) did not germinate adequately to support their inclusion in statistical analysis, and therefore, they were removed entirely from the dataset.

## Results

**Fig. 1:**
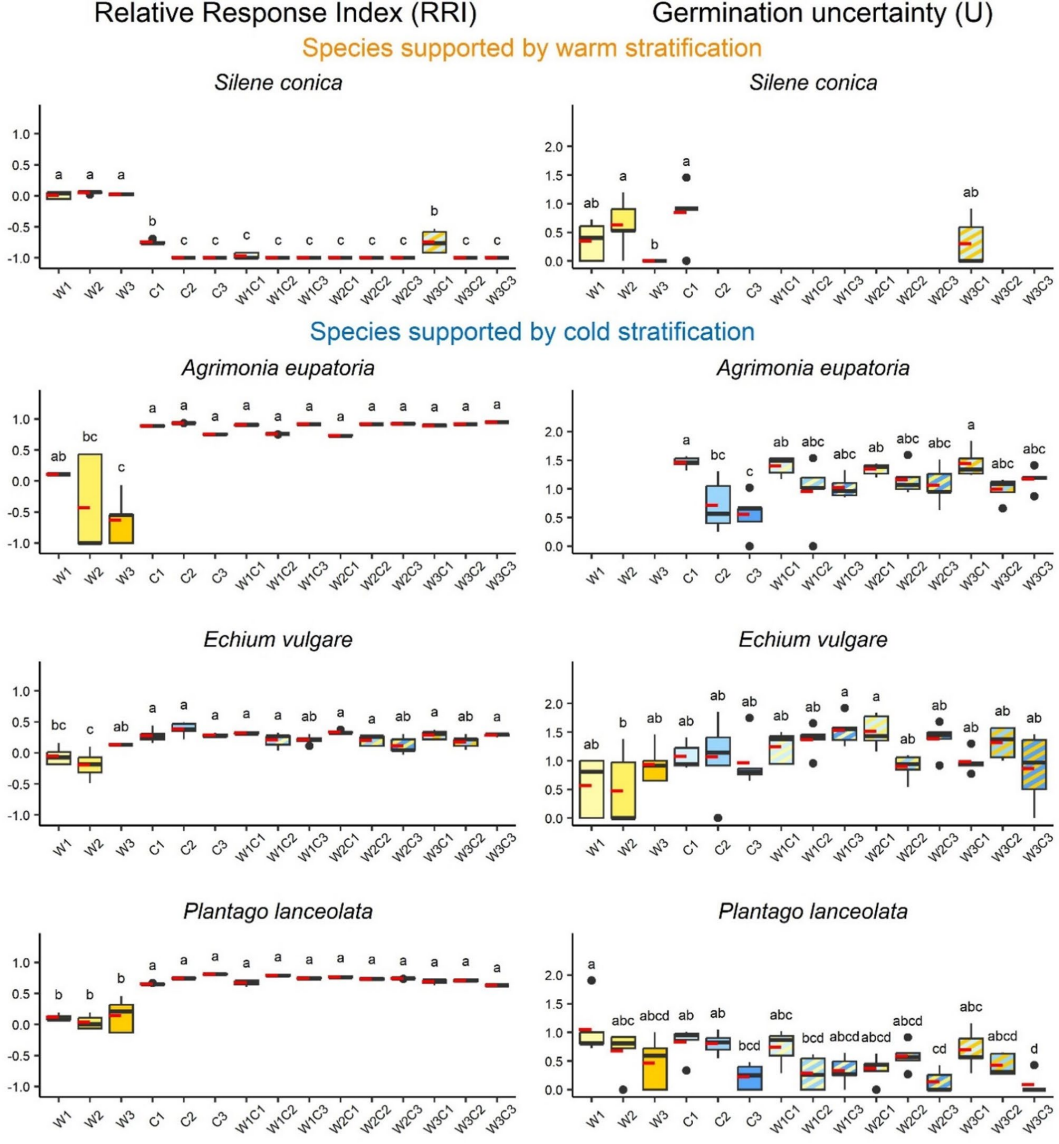
Response in the Relative response index (RRI, y axis of the left column) and Germination uncertainty (U, y axis of the right column) of four model species with clear responses to the stratification treatments. Medians are displayed with solid black lines and means with dashed red lines. Lower-case letters indicate significant differences between the treatments (GLM, p|<0.05). Treatment codes are abbreviated as follows: W1-3: 1–3 months of warm stratification, C1-3: 1–3 months of cold stratification. For the germination responses of all the studied species, see Table 1 and Fig. S1

Significant differences between stratification treatments were found in the majority of species (41 species), while treatments had no effect on germination success in three species (*Dianthus pontederae*, *Potentilla argentea*, *Teucrium chamaedrys*) (Fig. 1; Table 1, Fig. S1). Stratification treatments had significant effect on the U of 31 species and had no effect on the U of 13 species (Fig. 1, Table S3, Fig. S1).

Germination success of the controls of the four germination cycles was significantly different in 21 species and was similar in 23 species (Table S4, Fig. S2). Germination uncertainty (U) of the controls of the four germination cycles was significantly different in 8 species and was similar in all other species (36 species) (Table S5, Fig. S2).

**Table 1:**
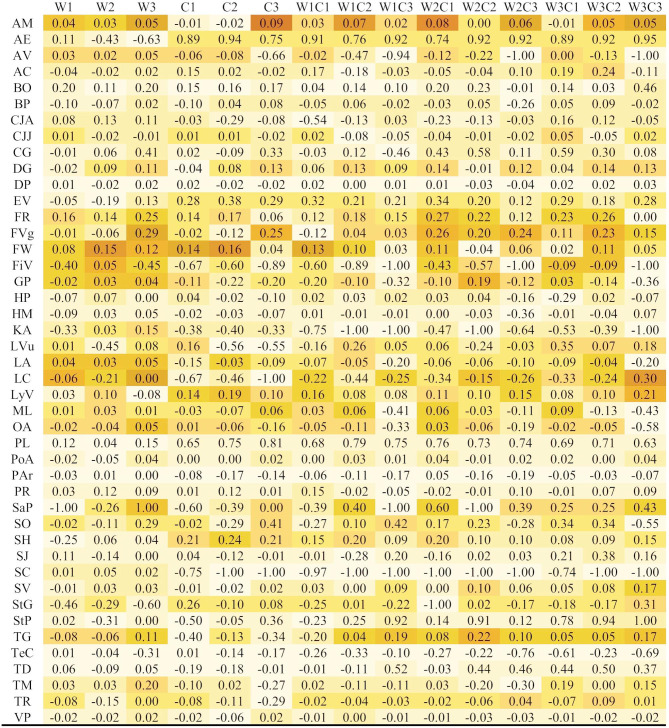
Mean relative response index (RRI) of 44 grassland species in the 15 stratification treatments

The darkening hue of cells indicate increasing mean RRI, with distinct hues representing groups corresponding to significant differences shown by letters in Fig. 1 and Fig. S1 (*p* ≤ 0.05). Species are abbreviated using the first letters of genus and species names, for the full list of abbreviations, please see Table S1. Treatment codes are abbreviated as follows: W1-3: 1–3 months of warm stratification, C1-3: 1–3 months of cold stratification. For further information, see Fig. S1

### Light requirements of species for germination

There was a proportion of species whose seeds started to germinate already in the dark in the cold wet stratification treatments. We noted these germination events as well, since these gave useful qualitative information on the light requirements of the species during germination. The 16 species which were able to germinate in dark were: *Anthyllis vulneraria*,* Dactylis glomerata*,* Dianthus pontederae*,* Echium vulgare*,* Festuca rupicola*,* Festuca vaginata*,* Festuca wagneri*,* Gypsophila paniculata*,* Hypochoeris maculata*,* Linum austriacum*,* Lotus corniculatus*,* Medicago lupulina*,* Potentilla argentea*,* Silene vulgaris*,* Trifolium montanum*, and *Trifolium repens*.

## Discussion

Four species showed clear responses to the warm and cold stratification treatments regardless of the length of these periods. These trends were not so obvious in other species. The effect of combined warm and cold stratification was greatly variable among the studied species. Germination uncertainty (U) generally remained high, even when germination success was elevated. This supports the idea that a bet-hedging strategy is commonly observed among Central European plant species, helping to minimize the risks during seedling establishment.

### Effect of the warm periods

A clear, strong positive response to warm stratification was found in *Silene conica*, while in other species there was no clear trend. *S. conica* expressed high germination success after warm stratification; however, one month of cold stratification drastically decreased its germination percentage, while longer cold periods completely blocked its germination. Increased germination through warm stratification and decreased germination due to cold stratification have also been observed in other *Silene* species typical of arid regions with hot, dry summers [54]. This pattern is characteristic of winter annuals [55, 56]. *S. conica* can clearly be categorized as an autumn germinating species; germinating in autumn favours its development during the favourable growing conditions of this period, characterised by increased precipitation, lower temperatures, as well as reduced interspecific competition [57]. Furthermore, despite the risk of frost damage, seedlings that overwinter in rosette form gain a competitive advantage as winter ends, allowing them to continue their life cycle as soon as temperatures begin to rise [16, 58].

In certain cases, such as in two *Festuca* species (*F. wagneri* and *F. rupicola*), the germination success associated with warm stratification treatments was reduced by the presence of a subsequent cold period. Applying a one-month-long cold stratification under similar conditions, Kövendi-Jakó et al. (2017) [46] also observed decreased germination success of *F. rupicola* and *F. vaginata*. However, in our study only longer cold periods had a negative effect on germination success, and in the case of *F. vaginata*, the effect was even positive. Besides, it seems that in *F. vaginata* the effects of warm- and cold stratifications are additive, or secondary dormancy does not occur and the seeds of the species tend to germinate especially after receiving at least a two-month-long warm stratification.

For two species with a wide distribution range (*Knautia arvensis*,* Linaria vulgaris*) we found that the effect of stratification treatments on germination differed from the responses found in other studies. In our study, warm stratification supported germination of *K. arvensis* more than cold stratification. In contrast, Vandvik & Vange (2003) [59] found that in northern populations of *K. arvensis* a two-month long cold stratification was the one that enhanced the seed germination. Similarly, *Linaria vulgaris* also showed differences in its germination success compared to the seeds originating from coastal dune habitats of the Baltic region [60]. In our case germination success decreased with the length of the cold period, while in the Baltic region highest germination of *L. vulgaris* was acquired after 5 months of cold stratification. These results highlight the importance of variability in germination requirements for species distribution: the variability in seed characteristics, including wide germination niches and diverse requirements for germination signals, supports the wide distribution of species [59, 60].

### Effect of the cold periods

Strong positive response to cold stratification, independently of its length, was found in three species: *Agrimonia eupatoria*, *Echium vulgare*, and *Plantago lanceolata*. The germination success of these species was consistently higher when cold stratification was applied compared to the treatments with only warm stratification. Warm stratification was either ineffective to break seed dormancy of these species or induced secondary dormancy. Our results are in agreement with other studies on *A. eupatoria* and *P. lanceolata*, which also found that the seeds of these species germinated better following exposure to cold periods or lower germination temperatures [61, 62]. Dormancy was successfully broken only after seeds were exposed to the optimal temperature conditions, i.e., optimal temperature range and duration. Without exposure to an optimal chilling temperature and duration, germination would not occur, posing a particular risk to of plant species relying on sexual reproduction via seeds. The optimal temperature range and duration are species-specific, varying from few days to several months [16, 44, 63]. In our case, a one-month-long cold period was sufficient to break dormancy in all three species. For *A. eupatoria*, shorter cold periods have also been reported, suggesting that the optimal duration of cold exposure required by this species ranges between two days [64] and ten days [62]. As the required cold period is so short, germination can already be induced during mild winter periods. Seedlings emerging during these periods may be exposed to the risk of frost damage, but overwintering individuals can benefit from favourable growing conditions even shortly after germination. However, to diminish the risks of winter frost periods, seed germination still remains scattered in time, assuring a fraction of seeds to be available for germination later during the growing period.

Germination follows a similar trend to the one described above in *Scirpoides holoschoenus* and *Lythrum virgatum* as well. In *L. virgatum* not only germination success, but germination synchrony as well was increased (indicated by the reduced germination uncertainty) by the presence of cold treatment. The strategy of this species differs from the majority of other species in our study; *L. virgatum* focuses on germination during favourable conditions rather than on risk-spreading by bet-hedging. However, considering that *L. virgatum* produces a large quantity of seeds, the persistence of the population can rely on few successfully surviving seeds and seedlings. Contrary to the above-mentioned species, where the length of cold period was not relevant, the length of the cold periods was important in *Stachys palustris* and *Silene vulgaris*, especially when preceded by warm stratification. In both cases it was already known that cold stratification increases germination success [46]but we also observed that in *S. palustris* the combination of warm and cold stratification treatments expressed an additive effect on germination.

In several species decreased germination was observed in response to prolonged cold periods (*Anthyllis vulneraria*, *Trifolium repens*) or in treatment combinations where prolonged cold periods followed warm periods (*Filipendula vulgaris*, *Medicago lupulina*, *Onobrychis arenaria*). Our result about *A. vulneraria* is in accordance with the results of a previous study showing that the species reacts well to shorter periods with cold temperature (1.5 months in 5°C) [65]. In *F. vulgaris* it was demonstrated that the species germinates better after warmer conditions during seed dormancy [66]. In four of the species listed above, we can assume that the responses were caused by complex mechanisms. These species belong to *Fabaceae* family, which often possess physical dormancy or combinational dormancy [17]. Due to their hard, water-impermeable seed coat they are physically dormant, and in some genera the development of the embryo is also physiologically inhibited. Seed germination of these species depends on both water uptake and the dormancy breaking process of the embryo. The sensitivity of the embryo to breaking dormancy is cyclical [18, 67]; when the embryo is not in a sensitive phase, water uptake may still occur, seeds may swell but germination does not take place. The mechanism works in the other way too: seeds may be in a sensitive state to break dormancy, but if water uptake is not possible, germination is hampered and seeds become insensitive again [18]. The role of this complex dormancy-breaking mechanism is similar to that of dormancy cycling in species with physiological dormancy: it initiates germination only when conditions are favourable for germination, seedling establishment, and survival [18].

The length of cold periods, as well as the length of warm periods, were also important for *Tragopogon dubius*. The effects of the warm and cold stratification treatments were additive in specific combinations: for successful germination, a shorter warm period had to be combined with a longer cold period. As the length of the warm period increased, the duration of the cold period required for successful germination decreased. Similar dormancy breaking mechanism was described for seeds possessing intermediate regular physiological dormancy (PD) but it was not known to be present in any of the genera of the *Asteraceae* family [36].

### Unaffected germination patterns

In *Dianthus pontederae*,* Potentilla argentea* and *Teucrium chamaedrys*, the stratification treatments did not result in significant differences in their RRIs. *D. pontederae* and *P. argentea* had a high germination success even in their controls, which means that either their dormancy was broken during the dry storage, or they have non-deep dormancy and can germinate under various settings as soon as the conditions become favourable. Besides, none of the stratification treatments induced secondary dormancy of the seeds. In a previous study we found similarly high germination success in *D. pontederae* [68]which supports the fact of *D. pontederae* seeds possessing non-deep dormancy and can germinate fast in response to favourable environmental conditions. In contrast *T. chamaedrys* germinated poorly even in controls. Considering that higher germination success for *T. chamaedrys* was reported earlier [69]we assume that our findings were related to the low seed viability in the used seed stock. This explanation can also be applied to *Galium verum*, one of the four species excluded from the analysis due to the lack of germination, as our previous study reported high germination capacity of the species [68]. Differences between the studies in species germination may be resulted from the inter-annual variability of environmental conditions, which influences growing conditions and thus the fitness of mother plants [70, 71]. Furthermore, while differences in germination between populations of a certain species are well recognized [36, 72, 73]within-population and even within-individual variability was also described [74]. These imply that the position of inflorescences on plants can determine the maturation period of their seeds, and so, the dormancy type of these seeds, even within the same pods [75, 76]. The within-individual bet-hedging strategy, i.e., variability in seed-maturation and dormancy breaking within the same seed cohort, serves the same adaptive function as within-population variability by spreading germination risk across time to enhance population persistence under unpredictable environmental conditions.

### Limitations of the study and further possibilities

Due to the high number of species and treatments and limited space availability in the growing chambers, germination of seeds took place in four consecutive cycles. The duration of the stratification treatments determined the germination cycle they were associated with, starting with the shorter treatments and progressing to the longer ones. For each germination event a separate control was included. Our results do not imply that a cycle effect was present during germination and we also reduced the effect of position within growing chamber by randomizing the position of Petri dishes during verifications. When we compared the germination success of dry-stored seeds, we found that in half of the species germination success decreased with the length of storage time, possibly as a result of viability-loss or of a prolonged after-ripening period causing secondary dormancy [34, 44, 77]. To account for the potential decreased germination of seeds, instead of raw germination percentage data, we used the relative response index (RRI), which takes into account the results of controls to calculate response to treatments. We are also aware that within-population and within-individual differences in seed-maturity could influence the results. We carefully mixed the available seed quantity of each species before assigning treatments to the seeds, which increased the chance that seeds with different characteristics were equally represented through the treatments. We are also aware that dry storage before the experiment could have a major impact on our results. However, as all seeds were stored under the same conditions before purchase and, in practice, restoration practitioners usually work with dry stored seeds, we believe that the used seed material was appropriate for our aims. We are also aware that in the present study only the effect of the temperature of the pre-germination period was studied on germination success and dynamics, while moisture content and temperature during germination was constant. In the future different environmental cues (moisture content, temperature during germination) should be studied both under controlled (laboratory) conditions, i.e. in similar experimental simulations, and under field conditions, to investigate species resilience to changing environmental conditions. Here we only studied germination capacity, but in future studies it would be important to evaluate the fitness of the germinated seedlings.

## Conclusions

Understanding environmental conditions that induce or hamper germination by inducing or releasing the dormancy of wild grassland species has practical implications. Seed-sowing based restoration practices should take into account the species-specific seed dormancy type and use appropriate sowing time combined with dormancy-breaking pre-treatments to increase restoration efficiency. Our results are of great importance in the context of climate change as restoration measures need to be flexibly adapted to the changing climate and so to the changing environmental conditions instead of following current environmental conditions. We found that in general the grassland species included in our study can cope with climate warming in the phenological stage of germination. Although their aboveground abundance might show great year-to-year variability, their seeds will be able to germinate thanks to the bet-hedging strategy which can support the persistence of the populations.

## Supplementary Information

Supplementary Material 1

## Data Availability

Data will be made available on request.
