# Supplementary material for: Effect of pre-germination temperature conditions on germination characteristics of temperate grassland species
Source: BMC Ecol Evol. 2025 Aug 14;25:82. doi: 10.1186/s12862-025-02424-5 (PMC12351819; doi:10.1186/s12862-025-02424-5)

Table S1. List of the studied species. Strikethrough letters indicate species which were excluded from statistical analysis due to the lack of germination.

| Species name | Abbreviation | Family | Growth form |
| --- | --- | --- | --- |
| *Achillea millefolium* L*.* | AM | Asteraceae | Perennial |
| *Agrimonia eupatoria* L*.* | AE | Rosaceae | *Perennial* |
| *Anthyllis vulneraria* L*.* | AV | Fabaceae | *Perennial* |
| *Astragalus cicer* L*.* | AC | Fabaceae | *Perennial* |
| *Betonica officinalis* L*.* | BO | Lamiaceae | *Perennial* |
| *Brachypodium pinnatum* (L.) P. Beauv. | BP | Poaceae | *Perennial* |
| *Centaurea jacea* ssp. *angustifolia* Gremli | CJA | Asteraceae | *Perennial* |
| *Centaurea jacea* ssp. *jacea* L*.* | CJJ | Asteraceae | *Perennial* |
| *Chrysopogon gryllus* (L.) Trin. | CG | Poaceae | *Perennial* |
| *Dactylis glomerata* L. | DG | Poaceae | *Perennial* |
| *Dianthus pontederae* A. Kern. | DP | Caryophyllaceae | *Perennial* |
| *Echium vulgare* L. | EV | Boraginaceae | ***Biannual*** |
| *Festuca rupicola* Heuff. | FR | Poaceae | *Perennial* |
| *Festuca vaginata* Waldst. et Kit. ex Willd. | FVg | Poaceae | *Perennial* |
| *Festuca wagneri* Degen, Thaisz et Flatt in Degen, Thaisz et Flatt | FW | Poaceae | *Perennial* |
| *Filipendula vulgaris* Moench | FiV | Rosaceae | *Perennial* |
| *~~Galium boreale~~* ~~L.~~ | ~~GB~~ | ~~Rubiaceae~~ | *~~Perennial~~* |
| *~~Galium verum~~* ~~L.~~ | ~~GV~~ | ~~Rubiaceae~~ | *~~Perennial~~* |
| *Gypsophila paniculata* L. | GP | Caryophyllaceae | *Perennial* |
| *Hypericum perforatum* L. | HP | Hypericaceae | *Perennial* |
| *Hypochoeris maculata* L. | HM | Asteraceae | *Perennial* |
| *Knautia arvensis* (L.) Coult. | KA | Dipsacaceae | *Perennial* |
| *Linaria vulgaris* Mill. | LVu | Scrophulariaceae | *Perennial* |
| *Linum austriacum* L. | LA | Linaceae | *Perennial* |
| *Lotus corniculatus* L. | LC | Fabaceae | *Perennial* |
| *Lythrum virgatum* L. | LyV | Lythraceae | *Perennial* |
| *Medicago lupulina* L. | ML | Fabaceae | ***Annual*** |
| *Onobrychis arenaria* (Kit.) DC. | OA | Fabaceae | *Perennial* |
| *Plantago lanceolata* L. | PL | Plantaginaceae | *Perennial* |
| *Poa angustifolia* L. | PoA | Poaceae | *Perennial* |
| *Potentilla argentea* L. | PAr | Rosaceae | *Perennial* |
| *Potentilla recta* L. | PR | Rosaceae | *Perennial* |
| *~~Salvia austriaca~~* ~~Jacq.~~ | ~~SA~~ | ~~Lamiaceae~~ | *~~Perennial~~* |
| *Salvia pratensis* L. | SaP | Lamiaceae | *Perennial* |
| *Scabiosa ochroleuca* L. | SO | Dipsacaceae | *Perennial* |
| *Scirpoides holoschoenus* (L.) Soják | SH | Cyperaceae | *Perennial* |
| *~~Schoenus nigricans~~* ~~L.~~ | ~~SN~~ | ~~Cyperaceae~~ | *~~Perennial~~* |
| *Senecio jacobaea* L. | SJ | Asteraceae | ***Biannual-Perennial*** |
| *Silene conica* L. | SC | Caryophyllaceae | ***Annual*** |
| *Silene vulgaris* (Moench) Garcke | SV | Caryophyllaceae | *Perennial* |
| *Stachys germanica* L. | StG | Lamiaceae | *Perennial* |
| *Stachys palustris* L. | StP | Lamiaceae | *Perennial* |
| *Teucrium chamaedrys* L. | TeC | Lamiaceae | *Perennial* |
| *Thymus glabrescens* Willd. | TG | Lamiaceae | *Perennial* |
| *Tragopogon dubius* Scop. | TD | Asteraceae | ***Biannual-Perennial*** |
| *Trifolium montanum* L. | TM | Fabaceae | *Perennial* |
| *Trifolium repens* L. | TR | Fabaceae | *Perennial* |
| *Verbascum phoeniceum* L. | VP | Scrophulariaceae | ***Biannual*** |

Table S2. Design of the experiment. Orange colour indicates warm stratification, blue colour indicates cold stratification, green colour indicates the germination experiment. Treatment codes are abbreviated as follows: W1-3: 1-3 months of warm stratification, C1-3: 1-3 months of cold stratification, K: control. Abbreviations: Mo.: Month.

| Code | Stratification | Mo. 1 | Mo. 2 | Mo. 3 | Mo. 4 | Mo. 5 | Mo. 6 | Mo. 7 |
| --- | --- | --- | --- | --- | --- | --- | --- | --- |
| K1 | 0 Mo.,  Control of cycle I |  |  |  |  |  |  |  |
| W1 | 1 Mo. warm |  |  |  |  |  |  |  |
| W2 | 2 Mo. warm |  |  |  |  |  |  |  |
| C1 | 1 Mo. cold |  |  |  |  |  |  |  |
| C2 | 2 Mo. cold |  |  |  |  |  |  |  |
| W1C1 | 1 Mo. warm +  1 Mo. cold |  |  |  |  |  |  |  |
| K2 | 0 Mo. ,  Control of cycle II |  |  |  |  |  |  |  |
| W3 | 3 Mo. warm |  |  |  |  |  |  |  |
| C3 | 3 Mo. cold |  |  |  |  |  |  |  |
| W1C2 | 1 Mo. warm +  2 Mo. cold |  |  |  |  |  |  |  |
| W2C1 | 2 Mo. warm + 1 Mo. cold |  |  |  |  |  |  |  |
| K3 | 0 Mo.,  Control of cycle III |  |  |  |  |  |  |  |
| W1C3 | 1 Mo. warm +  3 Mo. cold |  |  |  |  |  |  |  |
| W2C2 | 2 Mo. warm + 2 Mo. cold |  |  |  |  |  |  |  |
| W2C3 | 2 Mo. warm + 3 Mo. cold |  |  |  |  |  |  |  |
| W3C1 | 3 Mo. warm + 1 Mo. cold |  |  |  |  |  |  |  |
| W3C2 | 3 Mo. warm + 2 Mo. cold |  |  |  |  |  |  |  |
| K4 | 0 Mo.,  Control of cycle IV |  |  |  |  |  |  |  |
| W3C3 | 3 Mo. warm + 3 Mo. cold |  |  |  |  |  |  |  |

Table S3. Mean germination uncertainty (U) of the studied species in stratification treatments. The darkening hues of cells indicate increasing mean U, with distinct hues representing groups corresponding to significant differences shown by letters in Figure S1 (p ≤ 0.05). Species are abbreviated using the first letters of genus and species names, for the full list of abbreviations, please see Appendix 1. Treatment codes are abbreviated as follows: W1-3: 1-3 months of warm stratification, C1-3: 1-3 months of cold stratification. NA in the cells indicate the case when germination was not sufficient to calculate U.

|  | W1 | W2 | W3 | C1 | C2 | C3 | W1C1 | W1C2 | W1C3 | W2C1 | W2C2 | W2C3 | W3C1 | W3C2 | W3C3 |
| --- | --- | --- | --- | --- | --- | --- | --- | --- | --- | --- | --- | --- | --- | --- | --- |
| AM | 0.13 | 0.00 | 0.09 | 0.00 | 0.05 | 0.00 | 0.00 | 0.00 | 0.05 | 0.00 | 0.00 | 0.00 | 0.14 | 0.00 | 0.00 |
| AE | NA | NA | NA | 1.46 | 0.72 | 0.55 | 1.40 | 0.95 | 1.03 | 1.34 | 1.16 | 1.06 | 1.44 | 0.99 | 1.17 |
| AV | 0.43 | 0.44 | 0.13 | 0.00 | 0.17 | 0.46 | 0.00 | 0.14 | NA | 0.07 | 0.48 | NA | 0.94 | 0.75 | NA |
| AC | 1.25 | 1.13 | 1.15 | 0.59 | 0.23 | 0.00 | 0.09 | 0.14 | 0.09 | 0.00 | 0.00 | 0.71 | 0.38 | 0.16 | 0.25 |
| BO | 0.94 | 1.09 | 1.49 | 1.27 | 1.02 | 0.85 | 1.30 | 1.10 | 1.29 | 1.18 | 1.04 | 1.30 | 1.00 | 1.03 | 1.21 |
| BP | 1.24 | 1.66 | 1.32 | 1.23 | 0.96 | 0.49 | 1.30 | 0.93 | 0.66 | 1.27 | 1.23 | 1.36 | 0.79 | 1.37 | 1.35 |
| CJA | 1.00 | 1.13 | 0.45 | 0.58 | 0.51 | 0.58 | 0.33 | 0.16 | 0.34 | 0.53 | 0.50 | 0.33 | 0.78 | 1.00 | 0.16 |
| CJJ | 0.91 | 0.93 | 0.60 | 0.54 | 0.21 | 0.51 | 0.56 | 0.63 | 0.78 | 0.55 | 0.34 | 0.63 | 0.38 | 0.67 | 0.41 |
| CG | 1.09 | 1.11 | 0.73 | 1.15 | 0.86 | 0.78 | 0.91 | 0.37 | NA | 0.85 | 0.55 | 0.00 | 1.08 | 0.40 | 0.36 |
| DG | 0.91 | 1.23 | 1.02 | 1.13 | 1.08 | 0.56 | 1.07 | 1.07 | 0.66 | 0.97 | 1.64 | 0.97 | 1.04 | 1.54 | 1.13 |
| DP | 0.28 | 0.14 | 0.00 | 0.69 | 0.33 | 0.84 | 1.00 | 0.29 | 0.91 | 0.80 | 0.95 | 0.85 | 0.82 | 1.00 | 1.05 |
| EV | 0.56 | 0.47 | 0.94 | 1.08 | 1.06 | 0.97 | 1.24 | 1.37 | 1.53 | 1.51 | 0.90 | 1.39 | 0.98 | 1.32 | 0.86 |
| FR | 0.76 | 1.05 | 1.20 | 1.19 | 1.47 | 1.44 | 1.02 | 0.99 | 1.47 | 0.66 | 1.58 | 1.56 | 0.61 | 1.67 | 1.29 |
| FVg | 1.36 | 0.96 | 1.18 | 1.21 | 1.14 | 1.56 | 1.35 | 1.59 | 1.42 | 1.48 | 1.45 | 1.39 | 1.15 | 1.56 | 1.83 |
| FW | 0.97 | 1.02 | 0.78 | 0.98 | 1.27 | 1.87 | 1.28 | 1.28 | 1.28 | 1.26 | 1.77 | 1.70 | 0.94 | 2.07 | 1.59 |
| FiV | 0.34 | 0.71 | 0.35 | NA | NA | NA | NA | NA | NA | 0.73 | NA | NA | NA | NA | NA |
| GP | 0.50 | 0.23 | 0.26 | 0.37 | 0.34 | 0.44 | 0.16 | 0.58 | 0.27 | 0.55 | 0.91 | 1.30 | 0.91 | 0.66 | 0.83 |
| HP | 1.02 | 0.96 | 1.43 | 1.45 | 1.00 | 1.29 | 1.19 | 1.14 | 1.66 | 1.19 | 1.02 | 0.52 | 0.80 | 1.23 | 1.39 |
| HM | 0.80 | 0.88 | 0.99 | 1.19 | 0.72 | 0.29 | 0.88 | 0.18 | 0.40 | 0.11 | 0.27 | 0.90 | 0.56 | 0.84 | 0.37 |
| KA | 0.88 | 0.85 | 0.91 | 0.60 | 0.18 | NA | NA | NA | NA | NA | NA | NA | NA | NA | NA |
| LVu | 0.47 | 0.48 | 0.62 | 0.69 | 0.18 | 0.50 | 0.64 | 0.69 | 1.08 | 0.83 | 0.00 | 0.33 | 1.05 | 0.51 | 0.79 |
| LA | 1.07 | 0.94 | 1.22 | 0.55 | 0.17 | 1.01 | 0.27 | 0.39 | 0.83 | 0.82 | 1.20 | 1.03 | 0.94 | 0.57 | 1.45 |
| LC | 1.21 | 0.82 | 0.25 | 0.00 | 0.00 | 0.00 | 0.76 | 0.71 | 0.26 | 0.00 | 0.60 | 0.18 | 0.16 | 0.26 | 0.06 |
| LyV | 1.28 | 1.26 | 1.14 | 0.00 | 0.37 | 0.05 | 0.20 | 0.06 | 0.00 | 0.13 | 0.46 | 0.00 | 0.09 | 0.83 | 0.05 |
| ML | 0.28 | 0.29 | 0.05 | 1.27 | 1.00 | 0.00 | 0.15 | 0.13 | 0.25 | 0.28 | 1.01 | 1.10 | 0.00 | 0.30 | 0.19 |
| OA | 1.13 | 0.85 | 0.73 | 0.19 | 0.72 | 0.16 | 0.17 | 0.47 | 0.53 | 0.30 | 0.93 | 0.81 | 0.70 | 0.36 | 0.75 |
| PL | 1.05 | 0.67 | 0.46 | 0.83 | 0.81 | 0.23 | 0.74 | 0.28 | 0.33 | 0.37 | 0.58 | 0.14 | 0.69 | 0.43 | 0.09 |
| PoA | 1.03 | 1.08 | 0.96 | 0.93 | 0.95 | 1.62 | 0.77 | 1.04 | 1.58 | 1.16 | 1.37 | 1.41 | 1.08 | 1.31 | 1.27 |
| PAr | 1.01 | 1.00 | 0.58 | 0.88 | 0.96 | 0.31 | 0.94 | 0.50 | 1.08 | 0.55 | 1.07 | 1.38 | 0.94 | 1.17 | 1.08 |
| PR | 1.02 | 0.63 | 0.62 | 0.59 | 0.58 | 0.36 | 0.76 | 0.29 | 0.42 | 0.48 | 0.57 | 0.40 | 0.49 | 0.87 | 0.09 |
| SaP | NA | 0.18 | 0.16 | NA | NA | NA | NA | NA | NA | NA | NA | 0.20 | NA | 0.20 | NA |
| SO | 0.56 | 0.83 | 0.88 | 0.73 | 0.98 | 0.61 | 0.80 | 0.67 | 0.38 | 1.32 | 0.82 | 0.20 | 0.55 | 1.00 | 0.20 |
| SH | 0.79 | 1.30 | 1.05 | 1.10 | 0.91 | 1.15 | 1.11 | 0.84 | 0.95 | 0.86 | 0.65 | 0.50 | 0.96 | 0.80 | 0.70 |
| SJ | 0.82 | 0.49 | 0.74 | 0.52 | 0.00 | 0.26 | 0.61 | 0.34 | 0.14 | 0.58 | 0.59 | 0.52 | 0.50 | 0.00 | 0.31 |
| SC | 0.35 | 0.63 | 0.00 | 0.84 | NA | NA | NA | NA | NA | NA | NA | NA | 0.30 | NA | NA |
| SV | 0.86 | 0.79 | 0.58 | 0.66 | 0.62 | 0.41 | 0.36 | 0.34 | 0.42 | 0.37 | 0.44 | 0.39 | 0.65 | 0.87 | 0.75 |
| StG | 0.20 | 0.19 | NA | 0.48 | 0.55 | 0.30 | 0.00 | 0.52 | 0.50 | NA | 0.38 | 0.34 | 0.55 | 0.38 | 0.72 |
| StP | 1.05 | 0.82 | 0.93 | 0.62 | 0.79 | 1.32 | 1.12 | 1.39 | 0.83 | 0.89 | 0.78 | 0.68 | 0.50 | 1.04 | 1.01 |
| TeC | 0.40 | 0.64 | 0.58 | 0.52 | 0.30 | 0.65 | 0.55 | 0.32 | 0.54 | 0.83 | 0.77 | NA | NA | 0.93 | NA |
| TG | 1.00 | 0.87 | 0.22 | 0.18 | 0.68 | 0.13 | 0.21 | 0.28 | 0.18 | 0.08 | 0.09 | 0.31 | 0.25 | 0.29 | 0.14 |
| TD | 1.15 | 1.22 | 0.51 | 1.55 | 1.31 | 1.49 | 0.75 | 1.39 | 1.17 | 0.61 | 0.93 | 1.59 | 1.11 | 0.86 | 1.71 |
| TM | 0.59 | 0.47 | 0.07 | 0.99 | 0.14 | 0.00 | 1.06 | 0.00 | 0.00 | 0.94 | 0.28 | 0.34 | 1.07 | 0.65 | 0.42 |
| TR | 0.64 | 0.31 | 0.06 | 1.14 | 0.84 | 0.72 | 1.08 | 0.79 | 0.24 | 0.79 | 0.80 | 0.28 | 1.00 | 0.80 | 0.29 |
| VP | 1.05 | 0.50 | 0.05 | 0.05 | 0.05 | 0.05 | 0.34 | 0.21 | 0.13 | 0.00 | 0.42 | 0.11 | 0.29 | 0.24 | 0.24 |

Figure S1. Relative response index (RRI, y axis of first column) and Germination uncertainty (U), y axis of second column) of the species in the 15 treatments. Medians are displayed with solid black lines and means with dashed red lines. Lower-case letters indicate significant differences between the treatments (GLM, p<0.05). Treatment codes are abbreviated as follows: W1-3: 1-3 months of warm stratification, C1-3: 1-3 months of cold stratification.


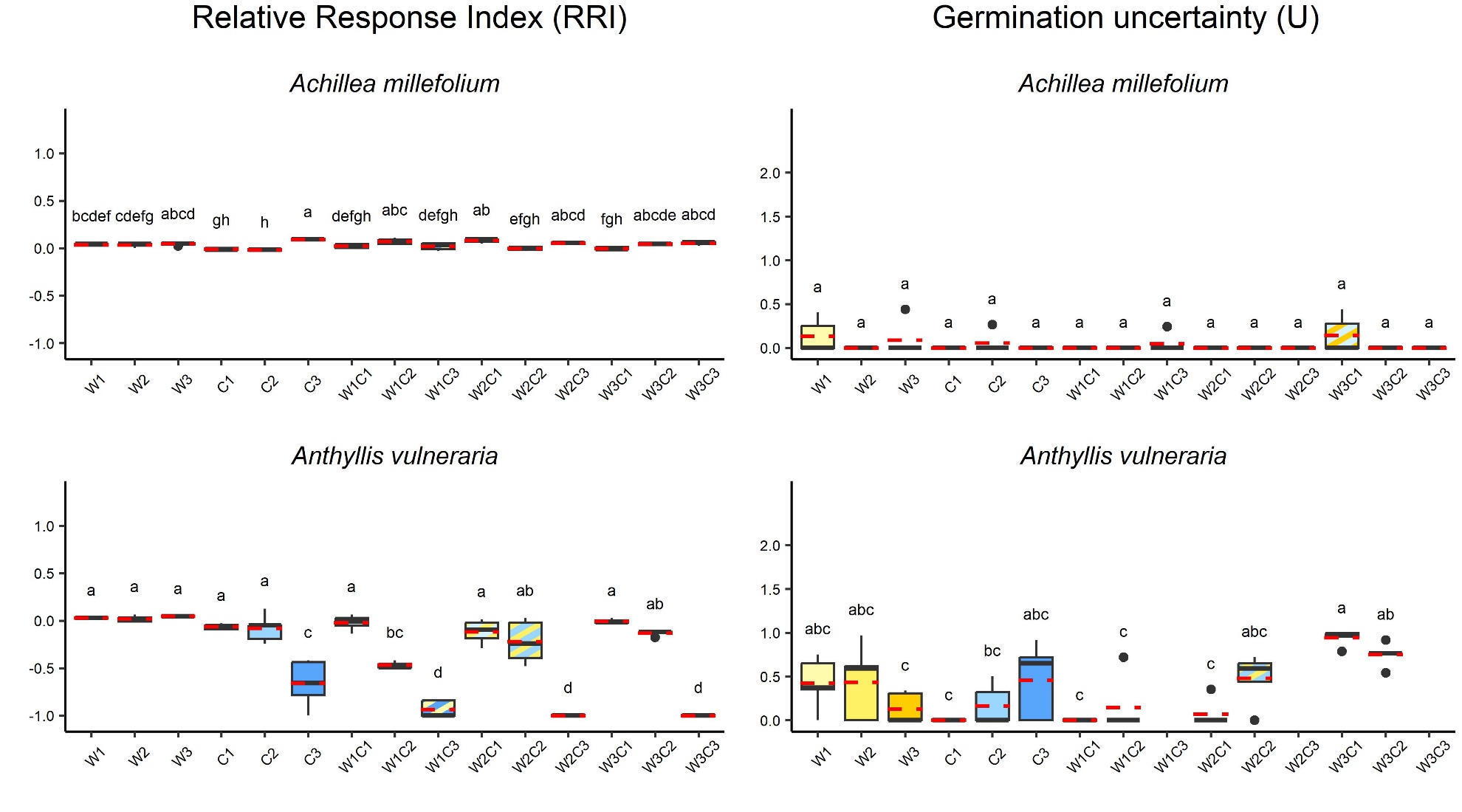

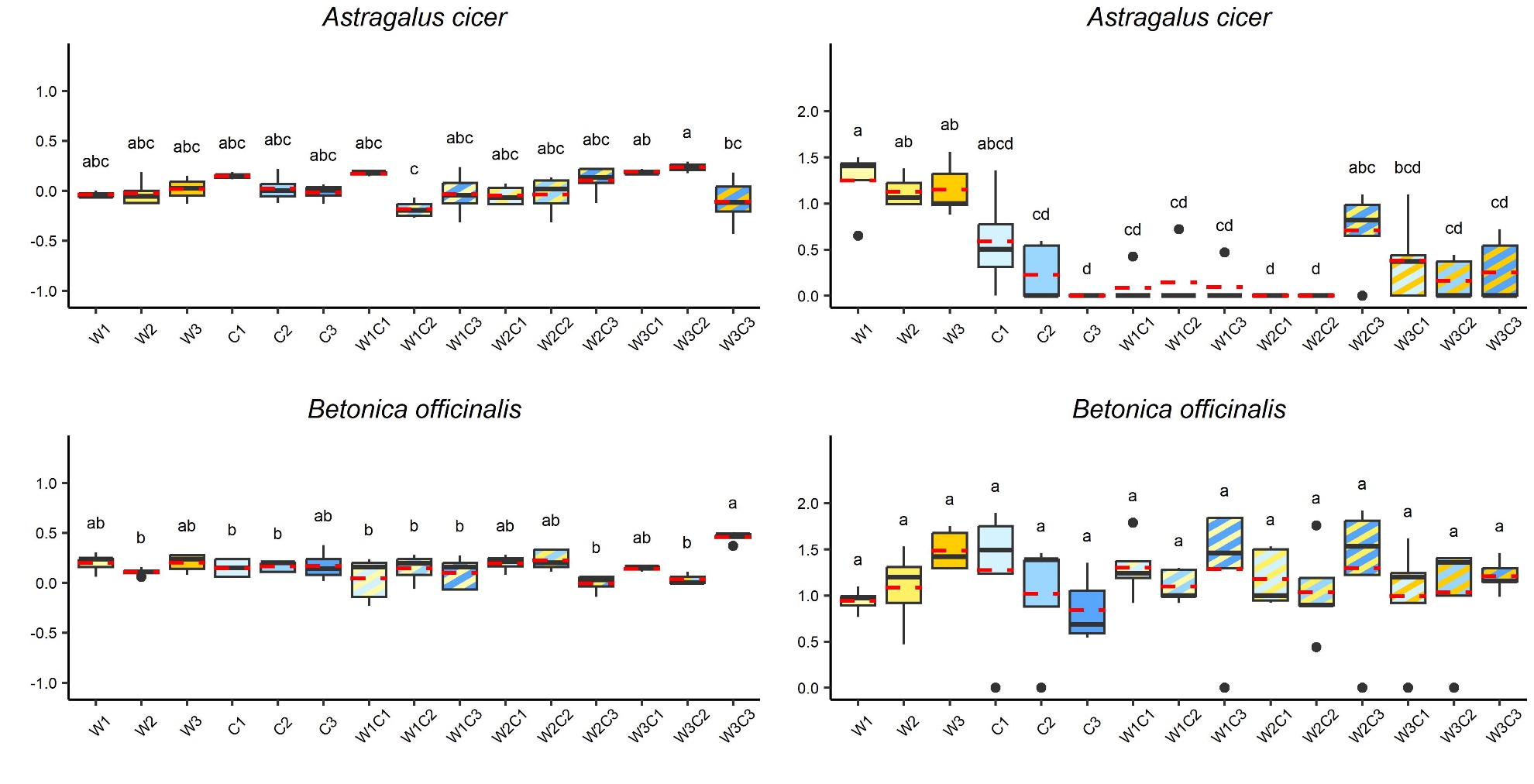

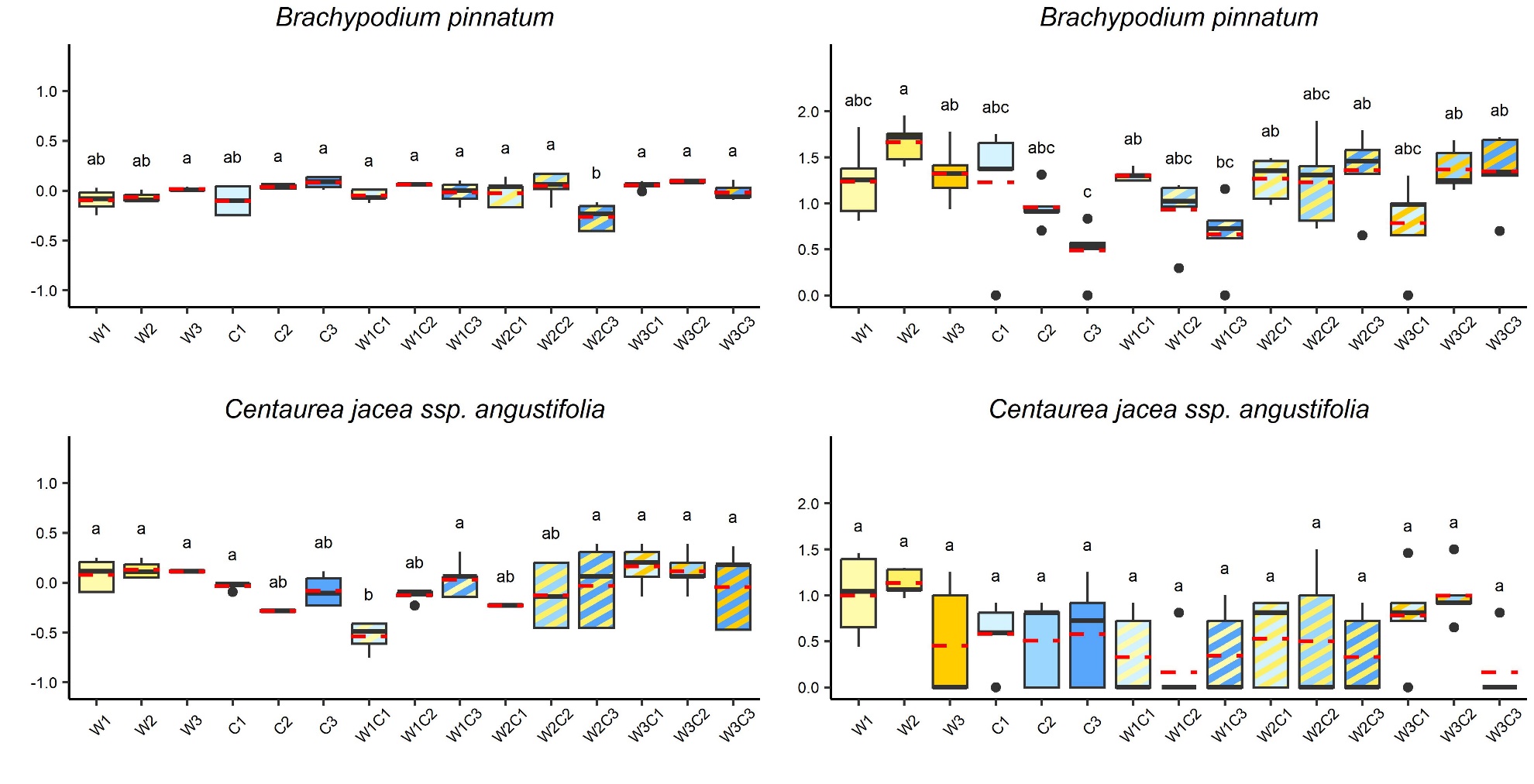

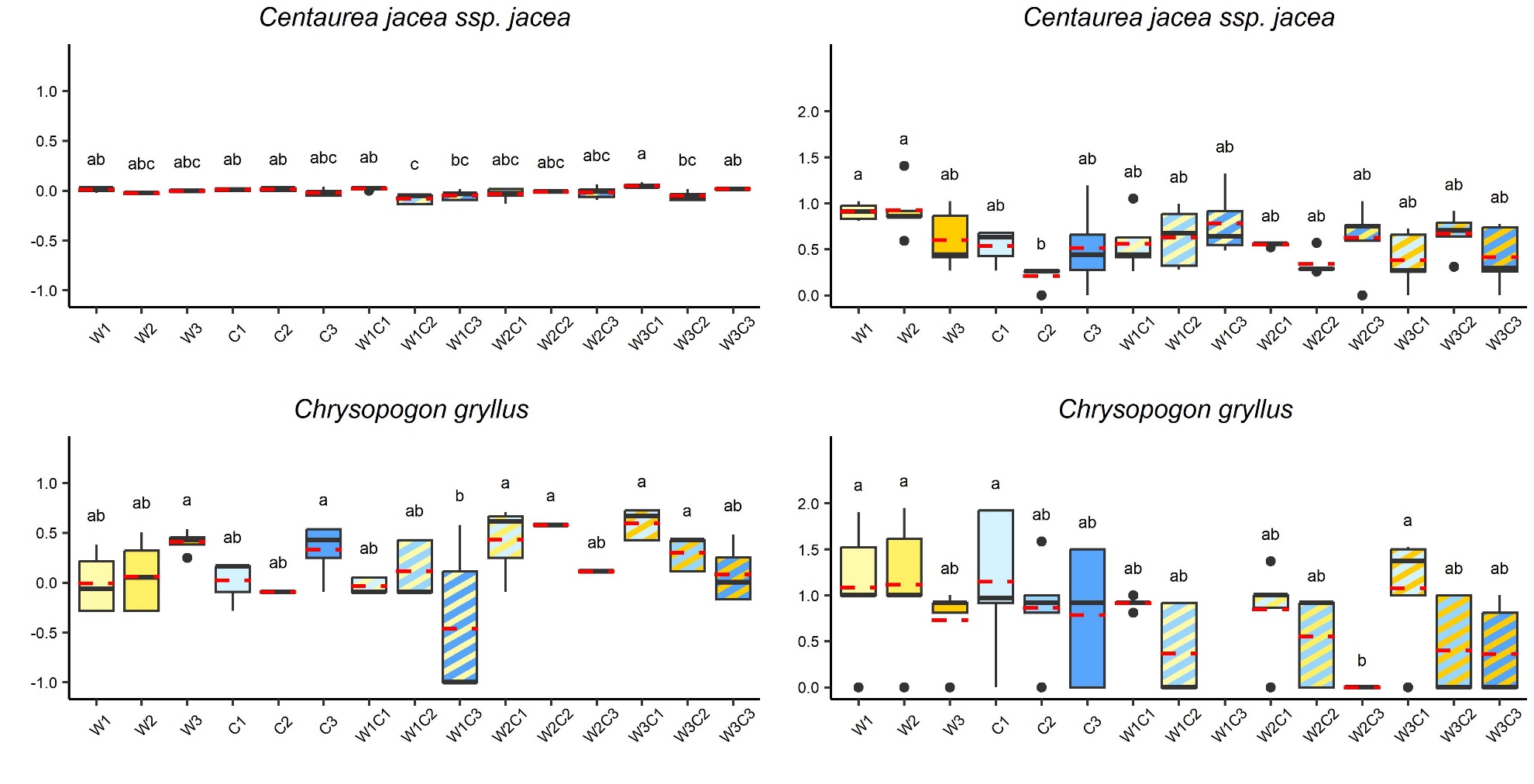

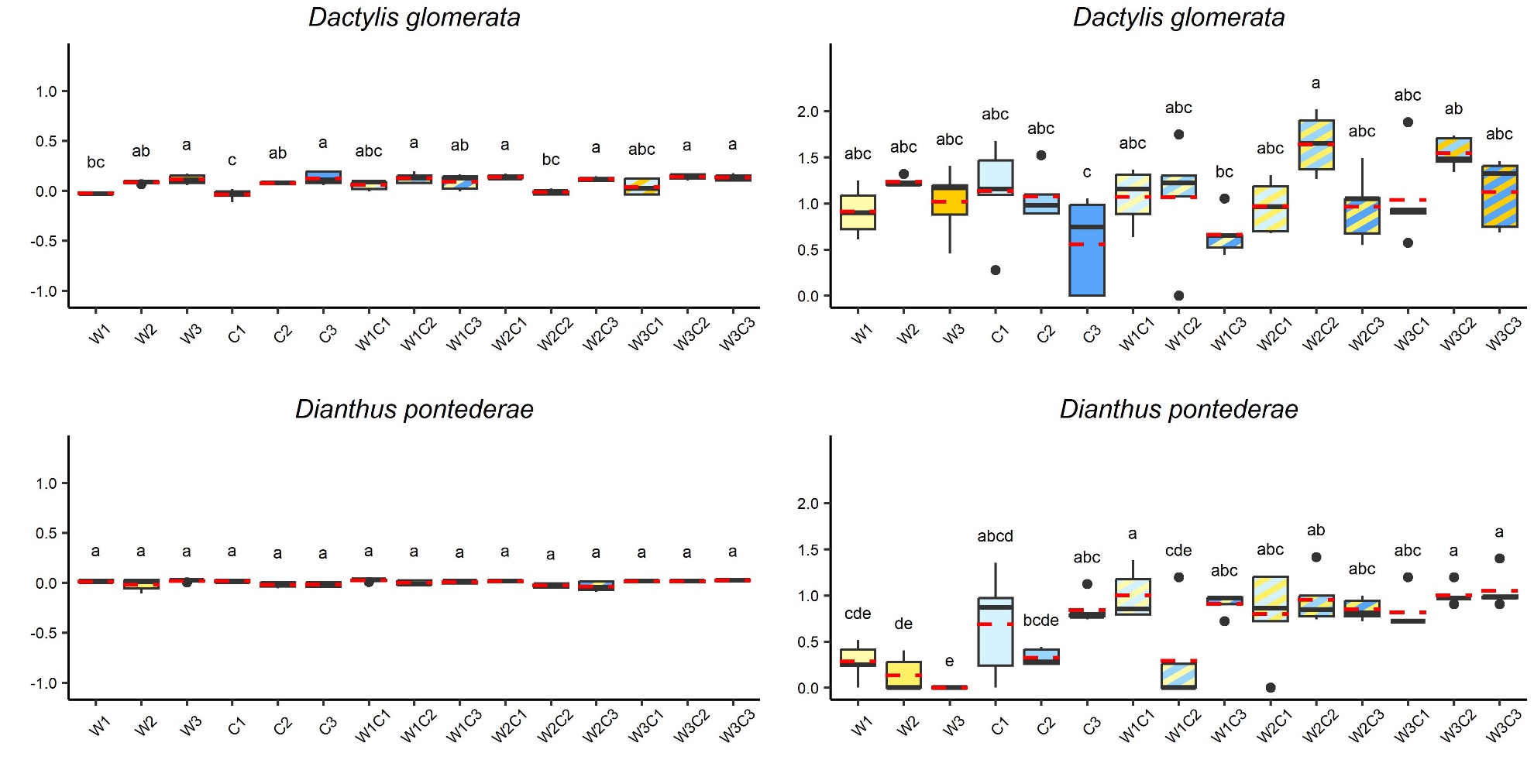

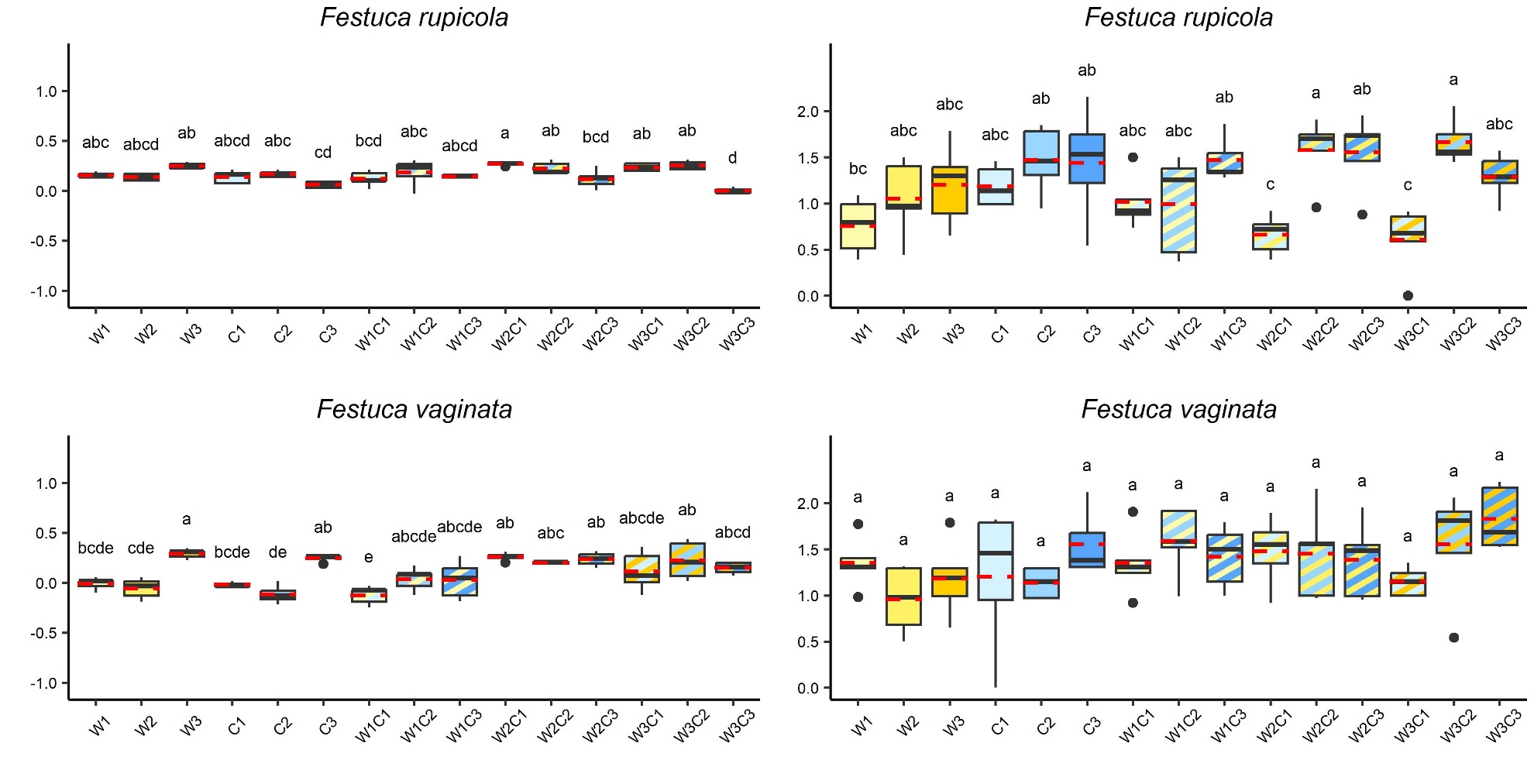

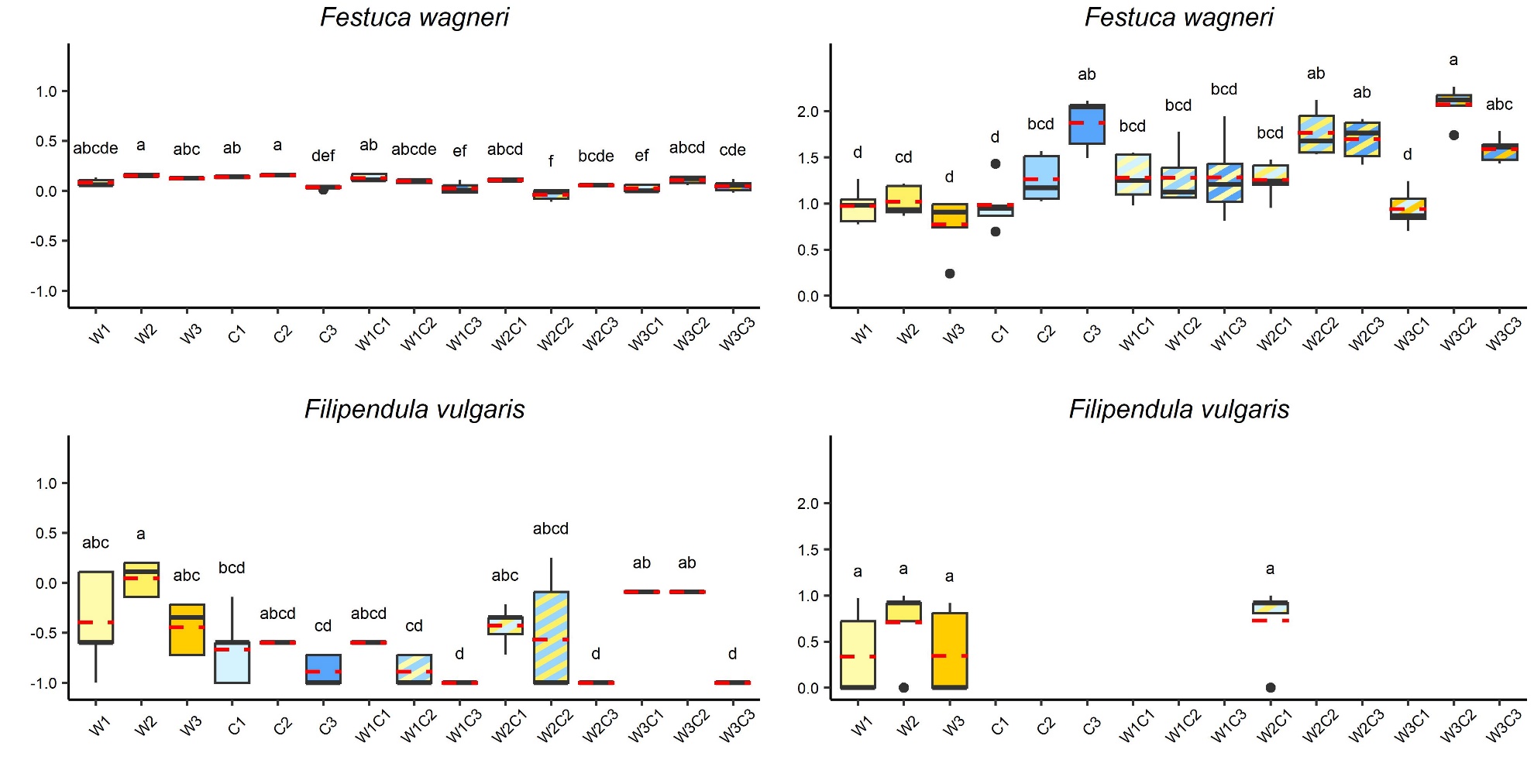

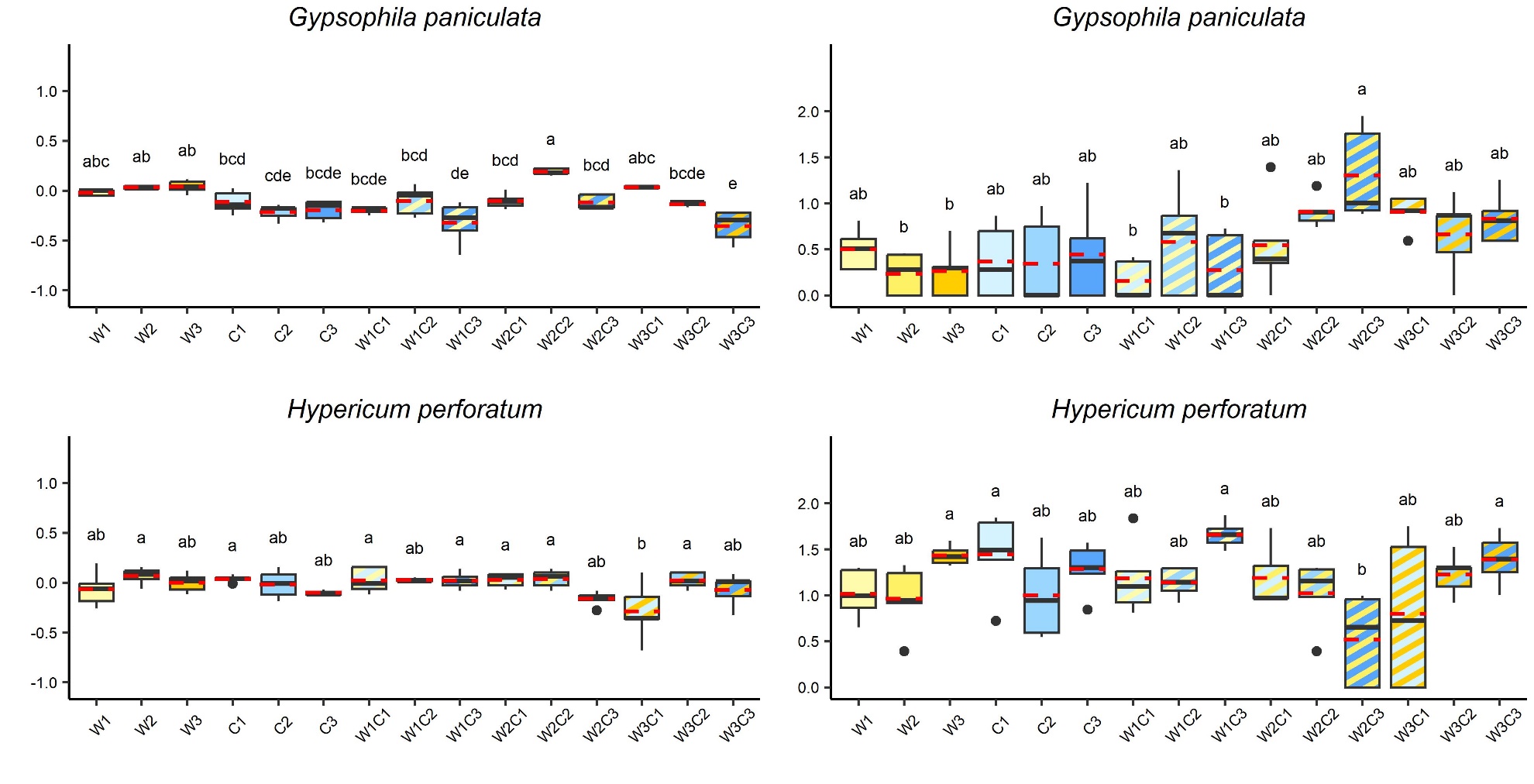

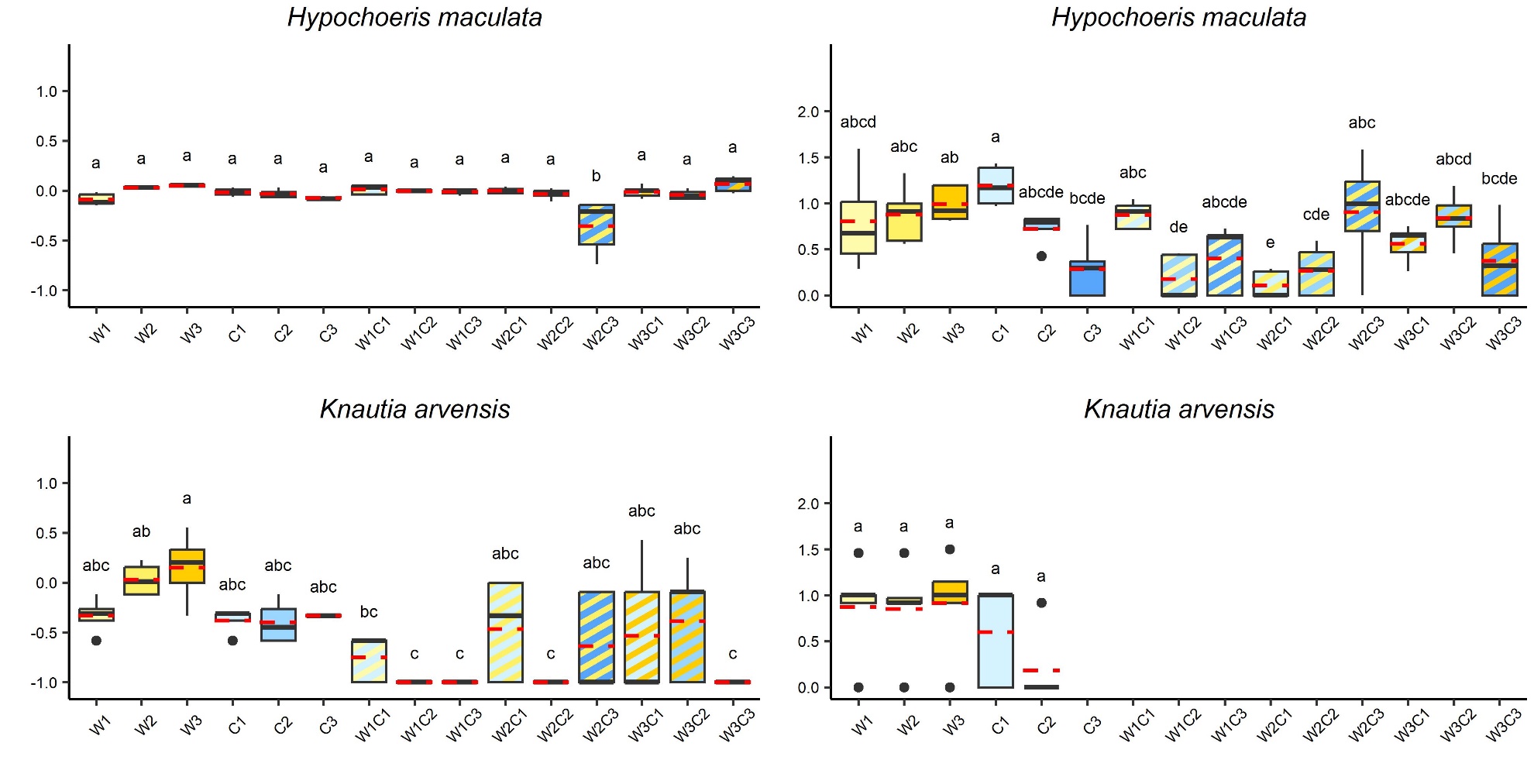

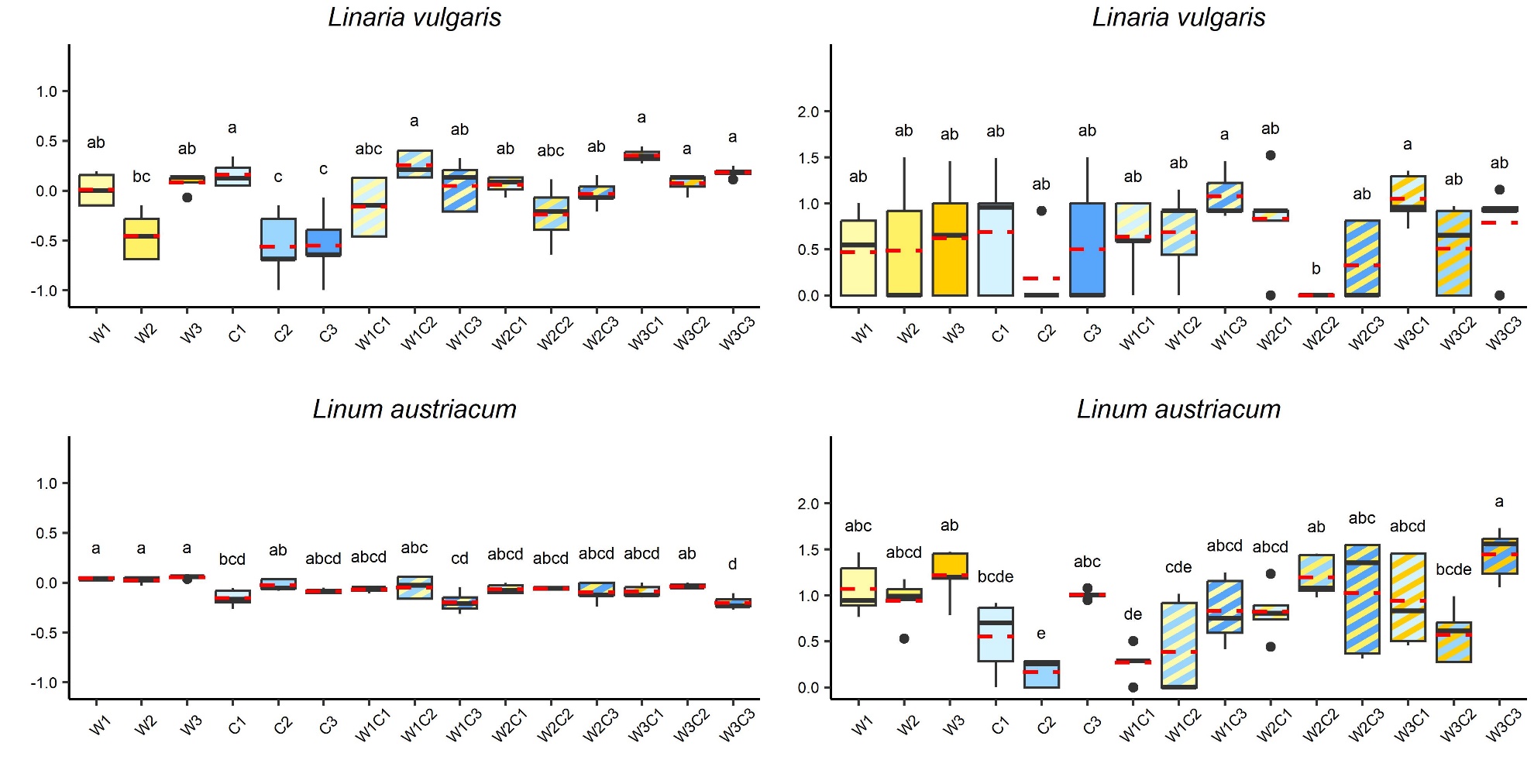

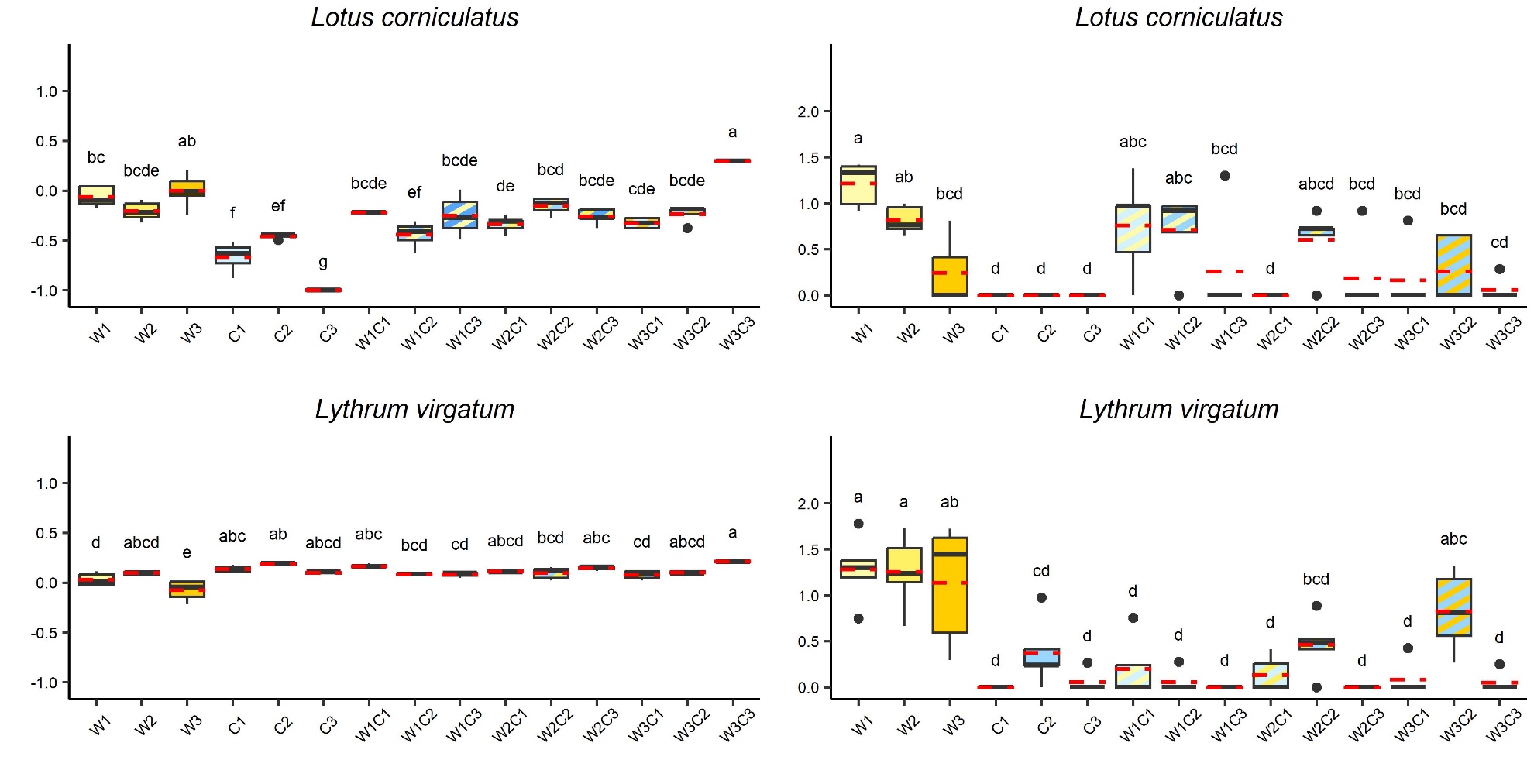

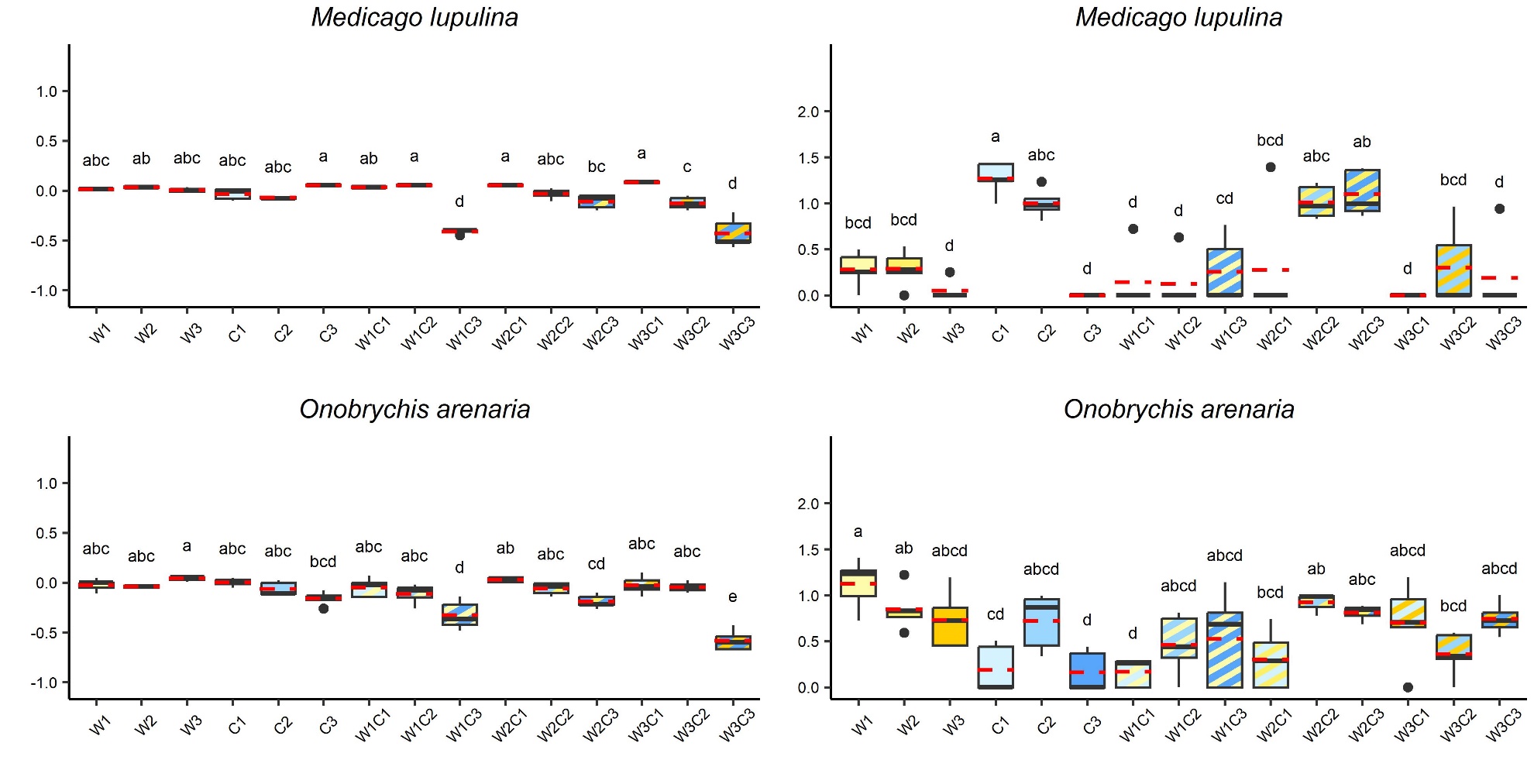

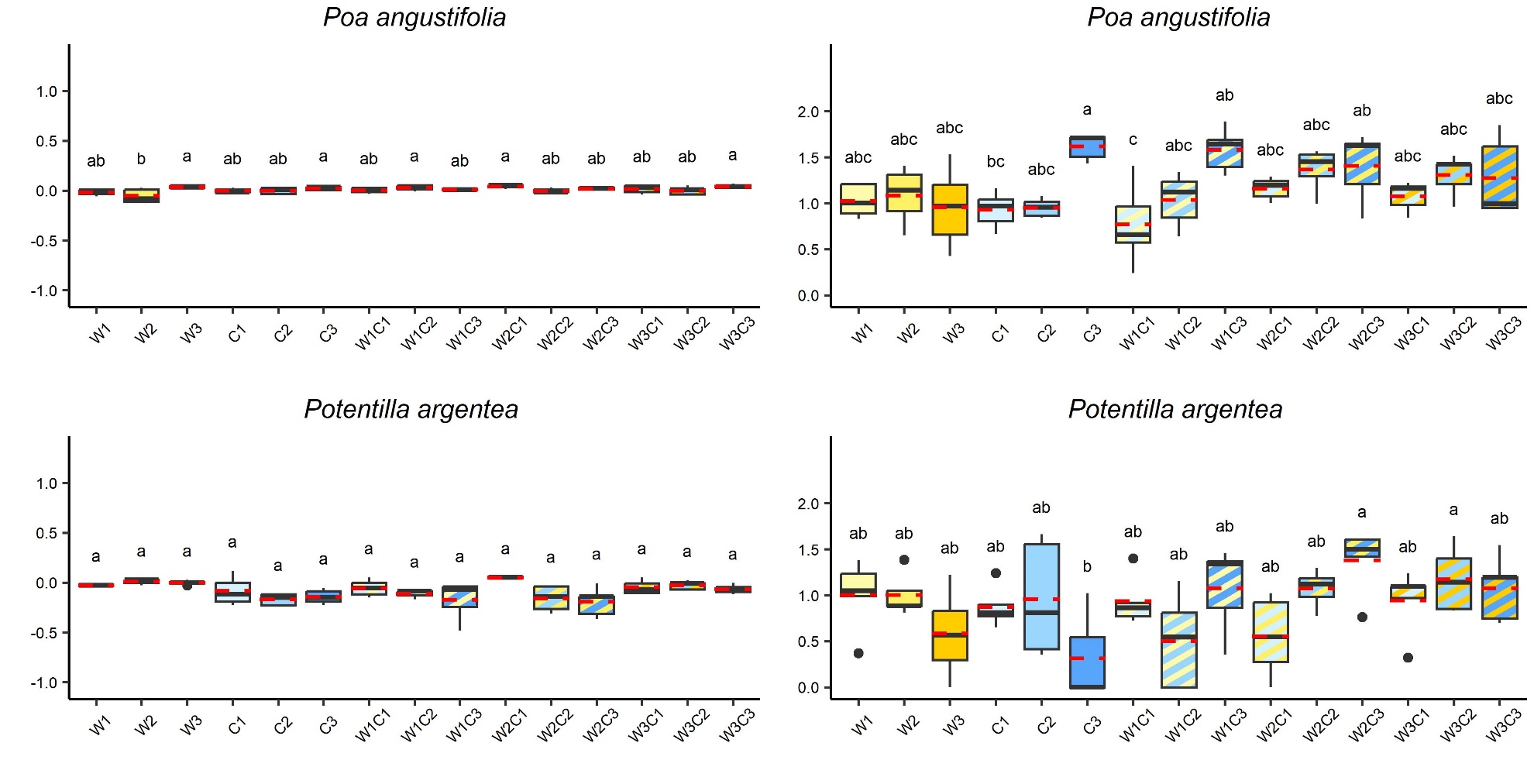

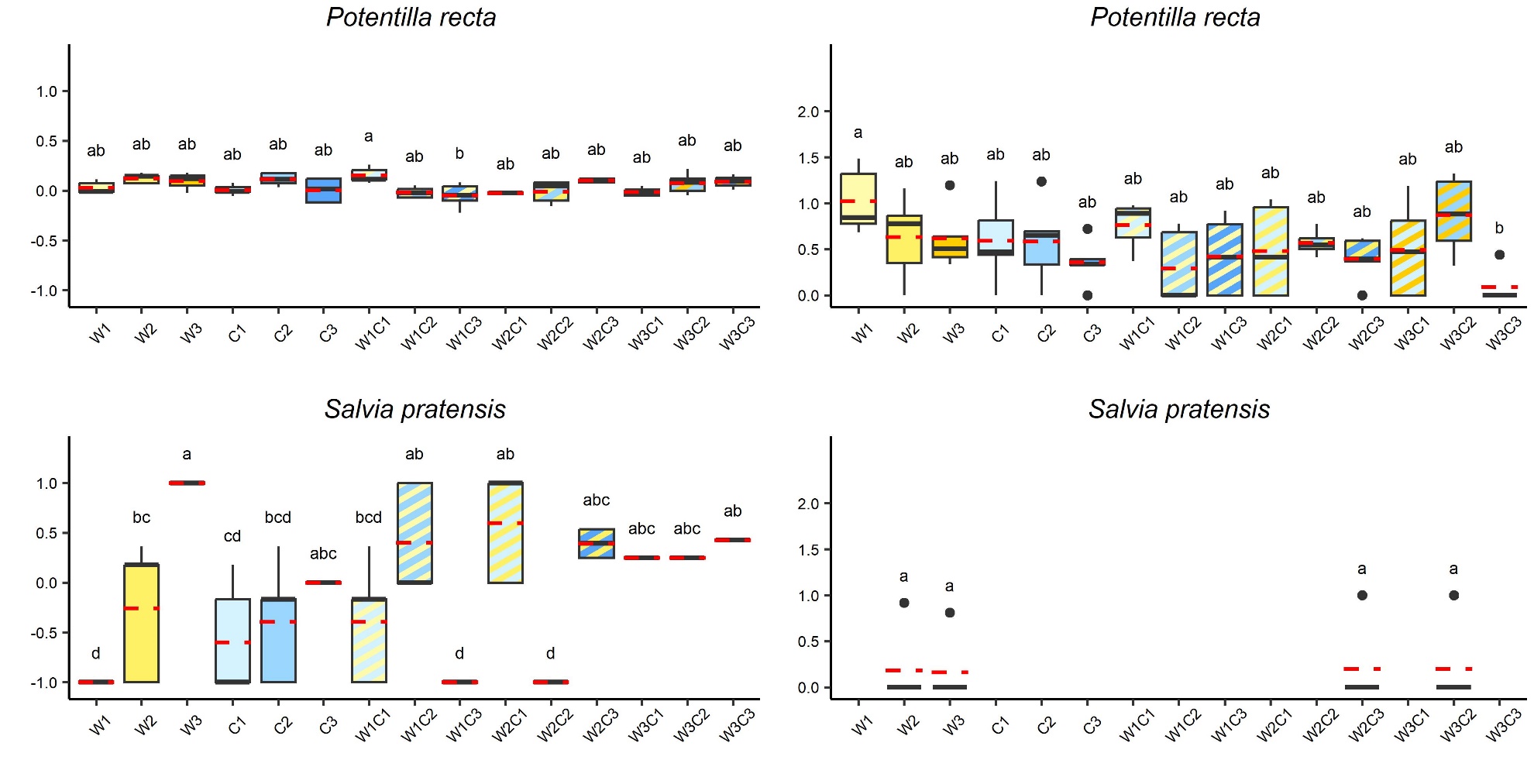

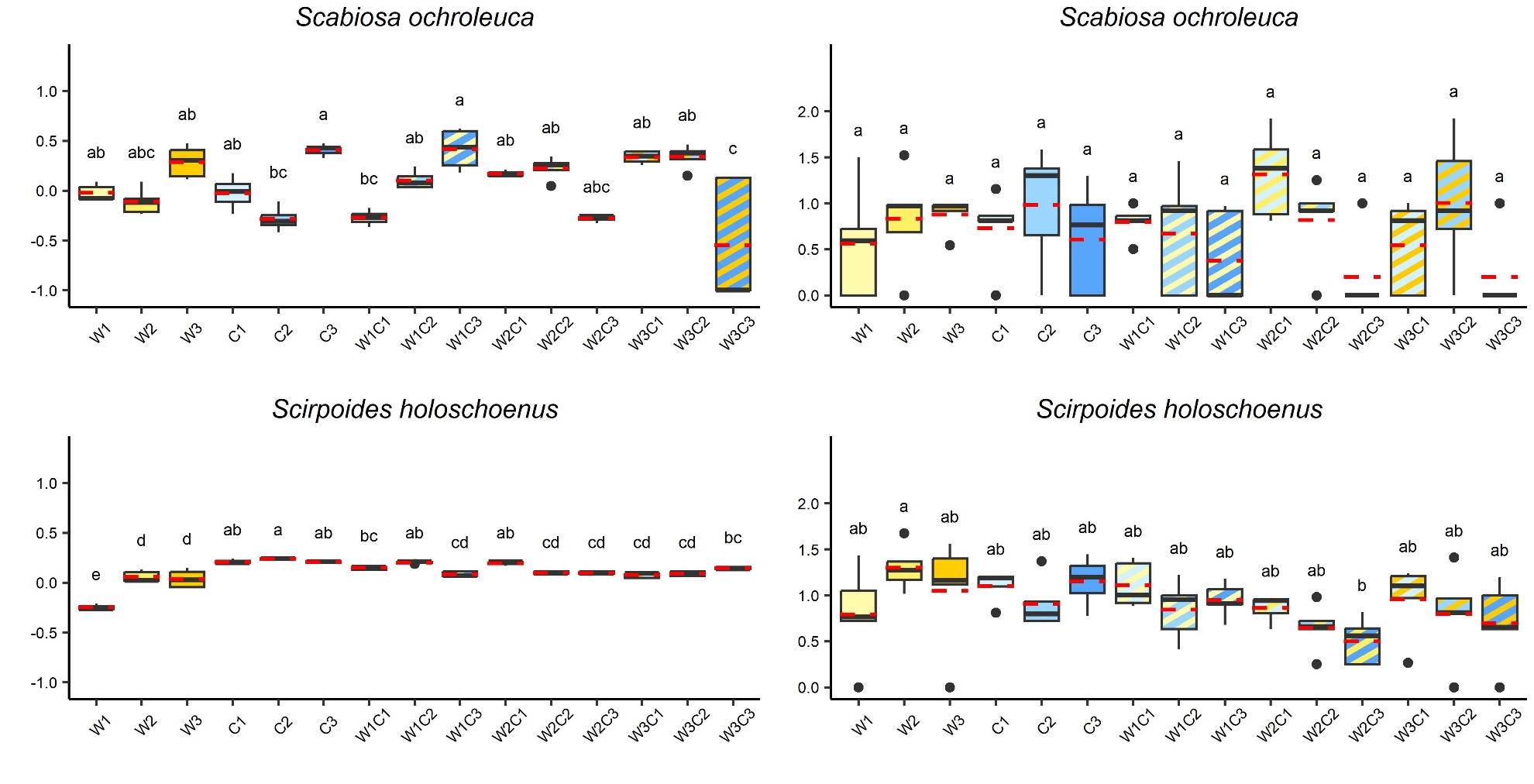

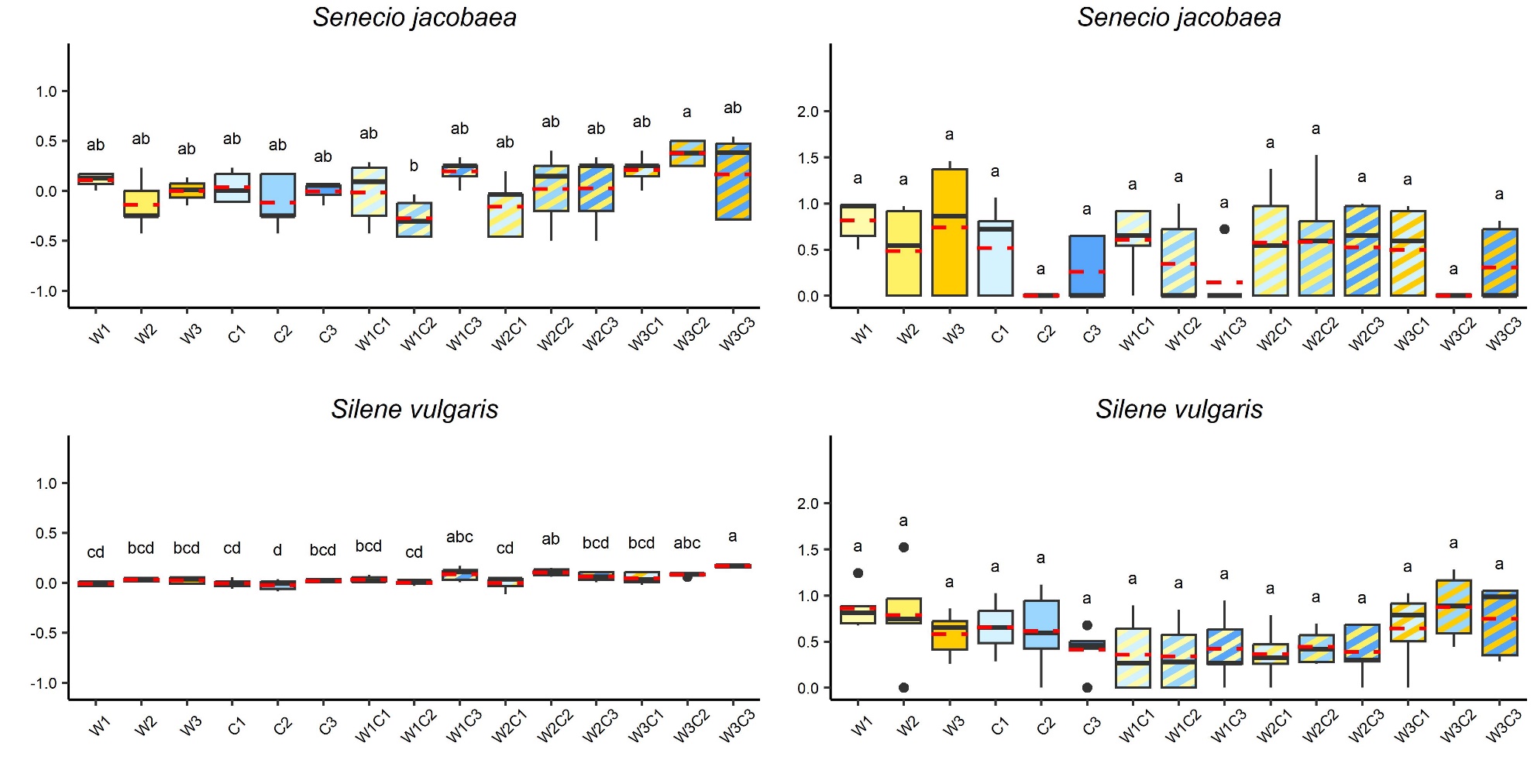

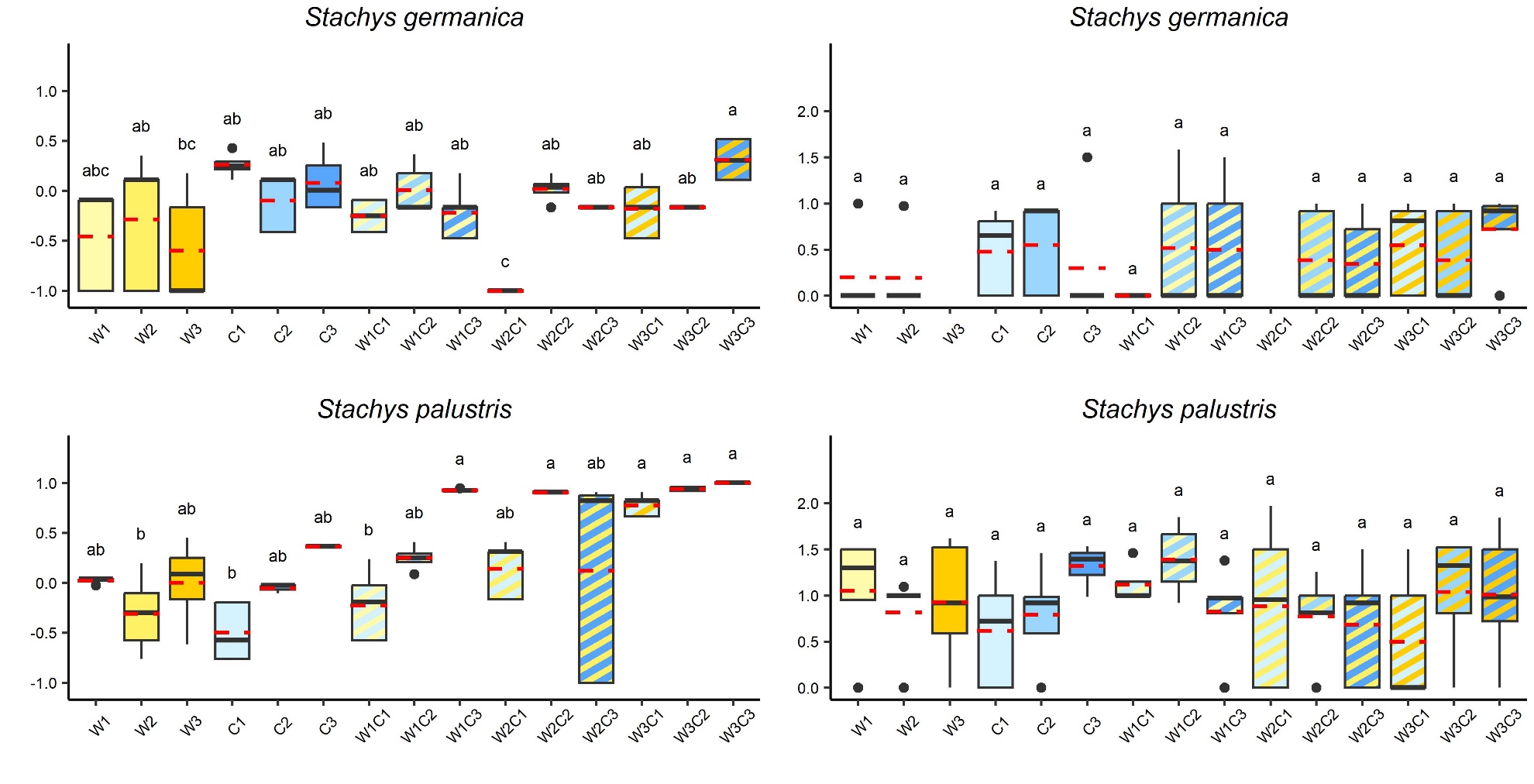

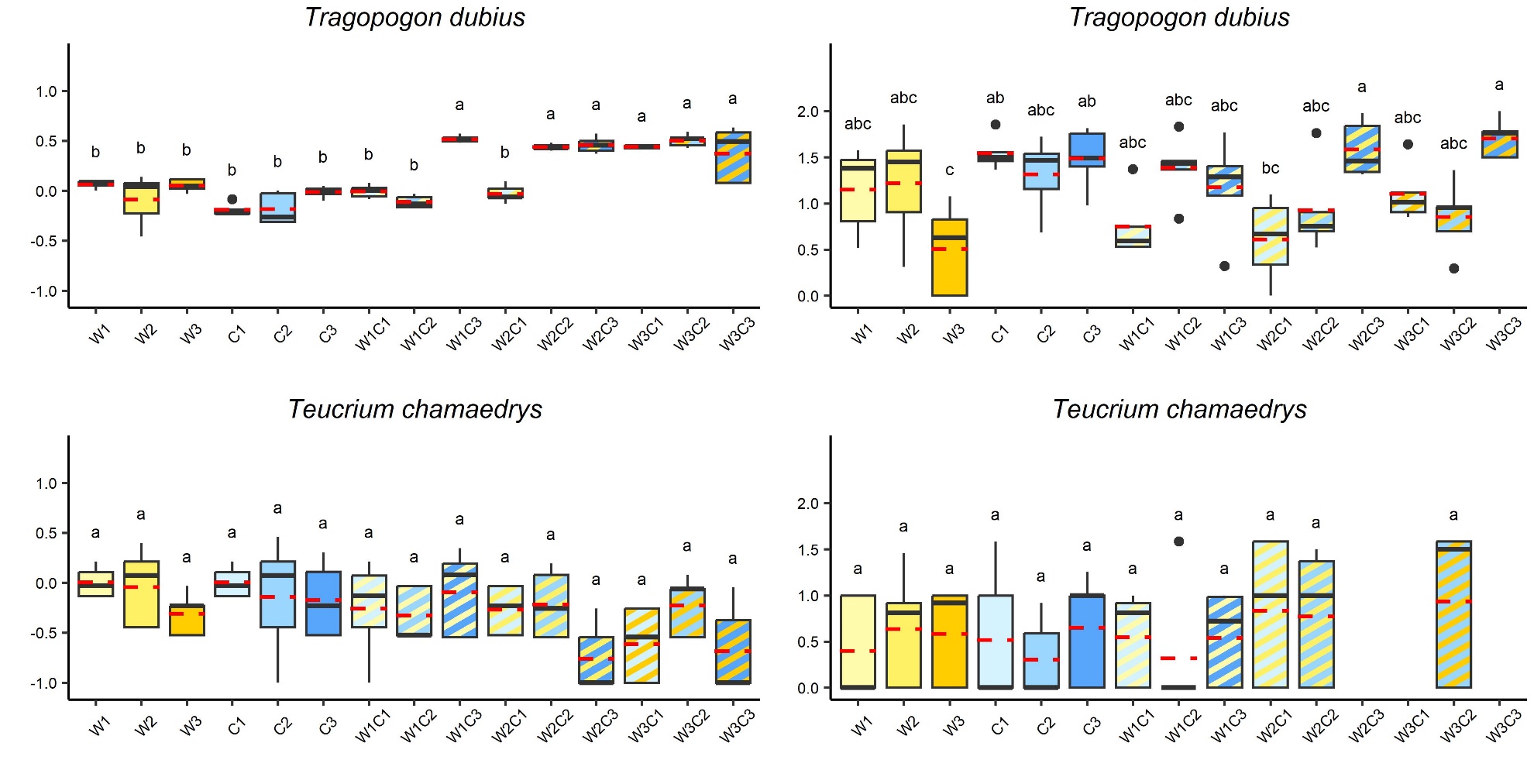

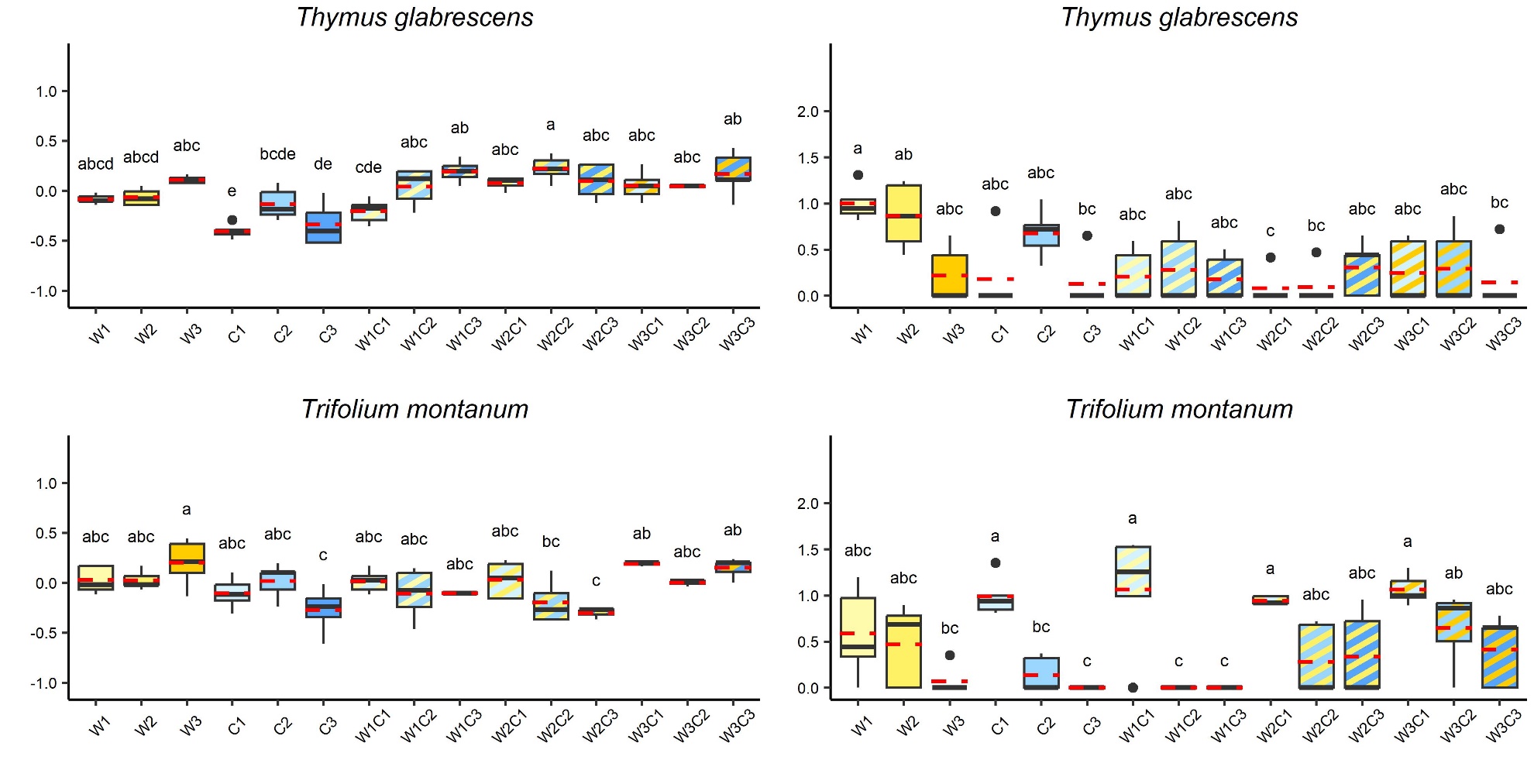

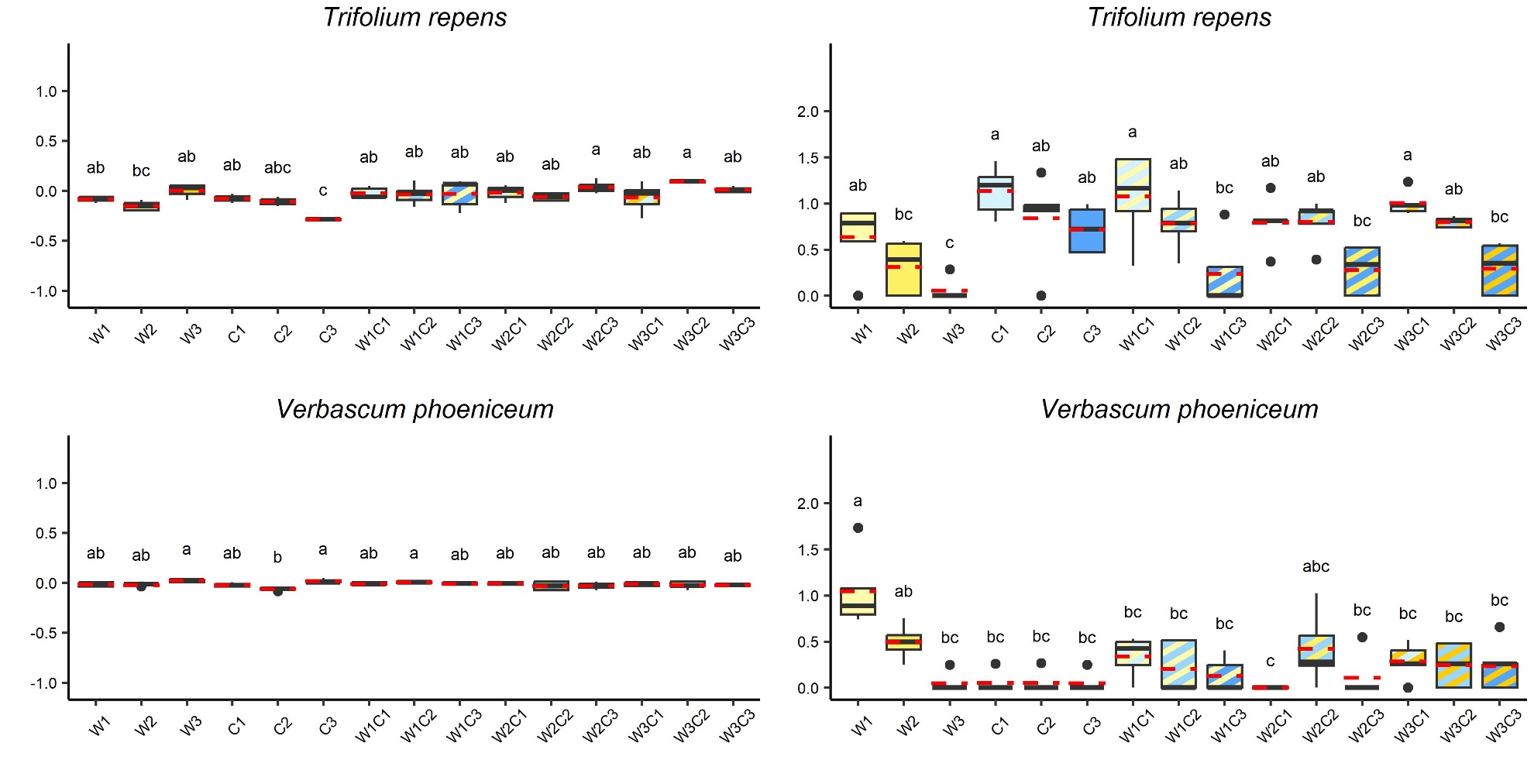


Table S4. Mean germination percentage (%) of the controls (K1-K4) of the four germination cycles. The darkening hue of cells indicate increasing mean germination success, with distinct hues representing groups corresponding to significant differences shown by letters in Figure S2 (*p* ≤ 0.05).

| Species name | Abbreviation | K1 | K2 | K3 | K4 |
| --- | --- | --- | --- | --- | --- |
| *Achillea millefolium* | AM | 91.20 | 80.00 | 89.60 | 88.00 |
| *Agrimonia eupatoria* | AE | 3.20 | 13.60 | 4.00 | 2.40 |
| *Anthyllis vulneraria* | AV | 52.80 | 58.40 | 45.60 | 39.20 |
| *Astragalus cicer* | AC | 35.79 | 41.60 | 30.89 | 30.40 |
| *Betonica officinalis* | BO | 32.00 | 27.20 | 32.00 | 18.40 |
| *Brachypodium pinnatum* | BP | 82.73 | 67.00 | 70.61 | 67.62 |
| *Centaurea jacea* ssp. *angustifolia* | CJA | 28.80 | 19.20 | 10.62 | 11.20 |
| *Centaurea jacea* ssp. *jacea* | CJJ | 88.00 | 88.80 | 81.60 | 73.60 |
| *Chrysopogon gryllus* | CG | 14.40 | 4.80 | 3.20 | 5.60 |
| *Dactylis glomerata* | DG | 80.80 | 68.00 | 68.80 | 61.60 |
| *Dianthus pontederae* | DP | 94.40 | 95.20 | 96.80 | 95.20 |
| *Echium vulgare* | EV | 23.20 | 18.40 | 25.60 | 21.60 |
| *Festuca rupicola* | FR | 64.95 | 49.87 | 52.40 | 61.81 |
| *Festuca vaginata* | FVg | 77.70 | 42.40 | 34.67 | 43.33 |
| *Festuca wagneri* | FW | 71.46 | 78.27 | 75.20 | 79.20 |
| *Filipendula vulgaris* | FiV | 16.00 | 24.80 | 4.80 | 5.60 |
| *Gypsophila paniculata* | GP | 80.00 | 70.40 | 56.00 | 44.00 |
| *Hypericum perforatum* | HP | 40.80 | 50.40 | 42.40 | 47.20 |
| *Hypochoeris maculata* | HM | 86.40 | 84.80 | 80.00 | 72.00 |
| *Knautia arvensis* | KA | 15.20 | 8.00 | 4.80 | 3.20 |
| *Linaria vulgaris* | LVu | 21.60 | 18.40 | 18.40 | 19.20 |
| *Linum austriacum* | LA | 89.60 | 88.80 | 92.00 | 84.00 |
| *Lotus corniculatus* | LC | 62.40 | 52.80 | 35.20 | 43.20 |
| *Lythrum virgatum* | LyV | 67.20 | 74.40 | 72.80 | 62.40 |
| *Medicago lupulina* | ML | 93.60 | 89.60 | 84.00 | 87.20 |
| *Onobrychis arenaria* | OA | 80.00 | 75.20 | 68.80 | 80.00 |
| *Plantago lanceolata* | PL | 13.60 | 10.40 | 13.60 | 20.80 |
| *Poa angustifolia* | PoA | 94.40 | 89.60 | 90.40 | 87.20 |
| *Potentilla argentea* | PAr | 76.00 | 76.00 | 68.80 | 80.80 |
| *Potentilla recta* | PR | 44.80 | 50.40 | 44.00 | 43.20 |
| *Salvia pratensis* | SaP | 5.60 | 0.00 | 2.40 | 1.60 |
| *Scabiosa ochroleuca* | SO | 58.51 | 24.00 | 11.88 | 10.00 |
| *Scirpoides holoschoenus* | SH | 61.60 | 65.60 | 80.00 | 72.80 |
| *Senecio jacobaea* | SJ | 20.00 | 21.60 | 12.00 | 7.20 |
| *Silene conica* | SC | 88.80 | 89.60 | 90.40 | 89.60 |
| *Silene vulgaris* | SV | 85.60 | 85.60 | 71.20 | 58.40 |
| *Stachys germanica* | StG | 9.60 | 5.60 | 11.20 | 6.40 |
| *Stachys palustris* | StP | 29.60 | 16.80 | 0.80 | 0.00 |
| *Teucrium chamaedrys* | TeC | 10.40 | 12.80 | 13.60 | 8.80 |
| *Thymus glabrescens* | TG | 58.40 | 37.60 | 25.60 | 16.00 |
| *Tragopogon dubius* | TD | 75.80 | 72.80 | 24.00 | 13.60 |
| *Trifolium montanum* | TM | 45.60 | 32.80 | 34.58 | 32.00 |
| *Trifolium repens* | TR | 76.80 | 72.00 | 63.20 | 61.60 |
| *Verbascum phoeniceum* | VP | 99.20 | 91.20 | 97.60 | 96.00 |

Table S5. Mean germination uncertainty (U) of the controls of the four germination cycles. The darkening hue of cells indicate increasing mean U, with distinct hues representing groups corresponding to significant differences shown by letters in Figure S2 (*p* ≤ 0.05). NA in the cells indicate the case when germination was not sufficient to calculate U.

| Species name | Abbreviation | K1 | K2 | K3 | K4 |
| --- | --- | --- | --- | --- | --- |
| *Achillea millefolium* | AM | 0.00 | 0.00 | 0.14 | 0.12 |
| *Agrimonia eupatoria* | AE | NA | NA | NA | NA |
| *Anthyllis vulneraria* | AV | 0.25 | 0.20 | 0.18 | 0.32 |
| *Astragalus cicer* | AC | 1.18 | 1.10 | 0.98 | 1.13 |
| *Betonica officinalis* | BO | 1.04 | 1.49 | 1.25 | 0.73 |
| *Brachypodium pinnatum* | BP | 1.28 | 0.95 | 1.35 | 1.03 |
| *Centaurea jacea* ssp. *angustifolia* | CJA | 0.55 | 0.96 | 0.74 | 0.77 |
| *Centaurea jacea* ssp. *jacea* | CJJ | 0.69 | 0.88 | 1.02 | 1.24 |
| *Chrysopogon gryllus* | CG | 0.50 | 0.38 | NA | 0.18 |
| *Dactylis glomerata* | DG | 1.28 | 0.93 | 1.28 | 1.00 |
| *Dianthus pontederae* | DP | 0.29 | 0.10 | 0.10 | 0.24 |
| *Echium vulgare* | EV | 0.48 | 0.76 | 0.86 | 0.48 |
| *Festuca rupicola* | FR | 1.05 | 0.61 | 0.97 | 0.93 |
| *Festuca vaginata* | FVg | 1.17 | 1.17 | 0.60 | 0.78 |
| *Festuca wagneri* | FW | 0.96 | 0.73 | 1.28 | 0.73 |
| *Filipendula vulgaris* | FiV | 0.48 | 0.88 | 0.00 | 0.16 |
| *Gypsophila paniculata* | GP | 0.36 | 0.11 | 0.55 | 0.97 |
| *Hypericum perforatum* | HP | 1.41 | 1.34 | 0.94 | 1.25 |
| *Hypochoeris maculata* | HM | 0.88 | 0.52 | 0.74 | 0.51 |
| *Knautia arvensis* | KA | 0.84 | 0.14 | 0.00 | NA |
| *Linaria vulgaris* | LVu | 0.89 | 0.38 | 0.13 | 0.19 |
| *Linum austriacum* | LA | 1.49 | 0.93 | 1.19 | 1.10 |
| *Lotus corniculatus* | LC | 0.76 | 0.16 | 0.40 | 0.77 |
| *Lythrum virgatum* | LyV | 1.19 | 1.36 | 1.21 | 1.32 |
| *Medicago lupulina* | ML | 0.10 | 0.00 | 0.00 | 0.16 |
| *Onobrychis arenaria* | OA | 0.56 | 0.18 | 0.55 | 0.49 |
| *Plantago lanceolata* | PL | 0.63 | 0.38 | 0.45 | 0.50 |
| *Poa angustifolia* | PoA | 1.08 | 1.06 | 0.54 | 0.67 |
| *Potentilla argentea* | PAr | 0.88 | 0.44 | 0.91 | 0.88 |
| *Potentilla recta* | PR | 0.76 | 0.67 | 0.29 | 0.44 |
| *Salvia pratensis* | SaP | NA | NA | NA | NA |
| *Scabiosa ochroleuca* | SO | 0.59 | 0.67 | 0.33 | 0.38 |
| *Scirpoides holoschoenus* | SH | 1.31 | 1.17 | 1.09 | 1.19 |
| *Senecio jacobaea* | SJ | 1.05 | 1.11 | 0.59 | 0.36 |
| *Silene conica* | SC | 0.23 | 0.18 | 0.72 | 0.52 |
| *Silene vulgaris* | SV | 0.81 | 0.62 | 1.28 | 1.20 |
| *Stachys germanica* | StG | 0.32 | 0.20 | 0.53 | 0.18 |
| *Stachys palustris* | StP | 1.36 | 1.35 | NA | NA |
| *Teucrium chamaedrys* | TeC | 0.55 | 0.68 | 0.98 | 0.40 |
| *Thymus glabrescens* | TG | 0.57 | 0.88 | 1.14 | 0.70 |
| *Tragopogon dubius* | TD | 1.45 | 1.32 | 0.99 | 0.87 |
| *Trifolium montanum* | TM | 0.26 | 0.18 | 0.47 | 0.18 |
| *Trifolium repens* | TR | 0.57 | 0.41 | 0.85 | 0.62 |
| *Verbascum phoeniceum* | VP | 0.26 | 0.30 | 0.84 | 0.31 |

Figure S2. Differences in the Germination success (%, y axis of first and second column) and Germination uncertainty (U, y axis of third and fourth column) of the 44 studied species between the controls of the four germination cycles. Medians are displayed with solid black lines and means with dashed red lines. Where U could be not calculated boxes of the given treatment or even species are not shown (see Table S5). Lower-case letters indicate significant differences between the treatments (Tukey-test, p<0.05).


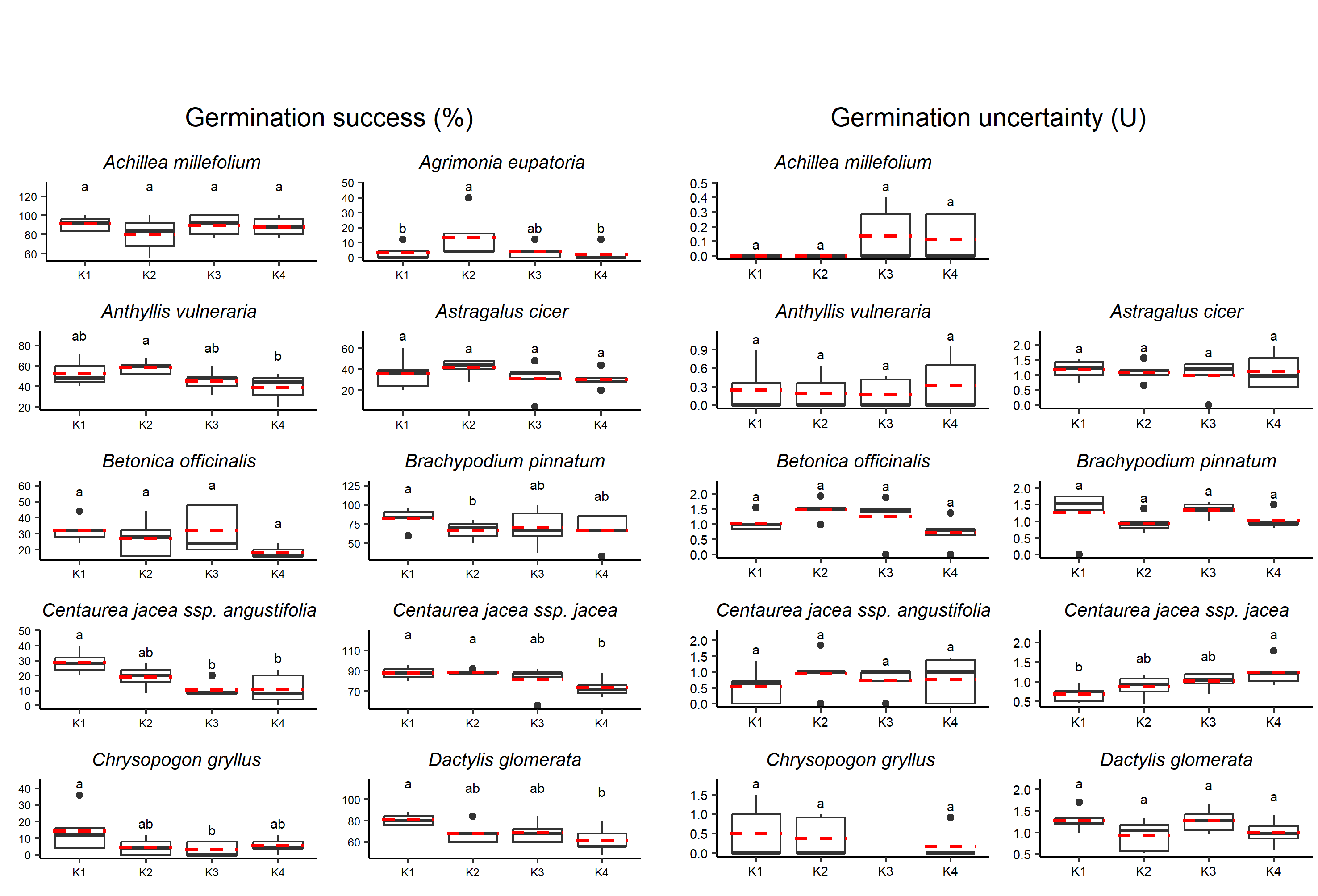

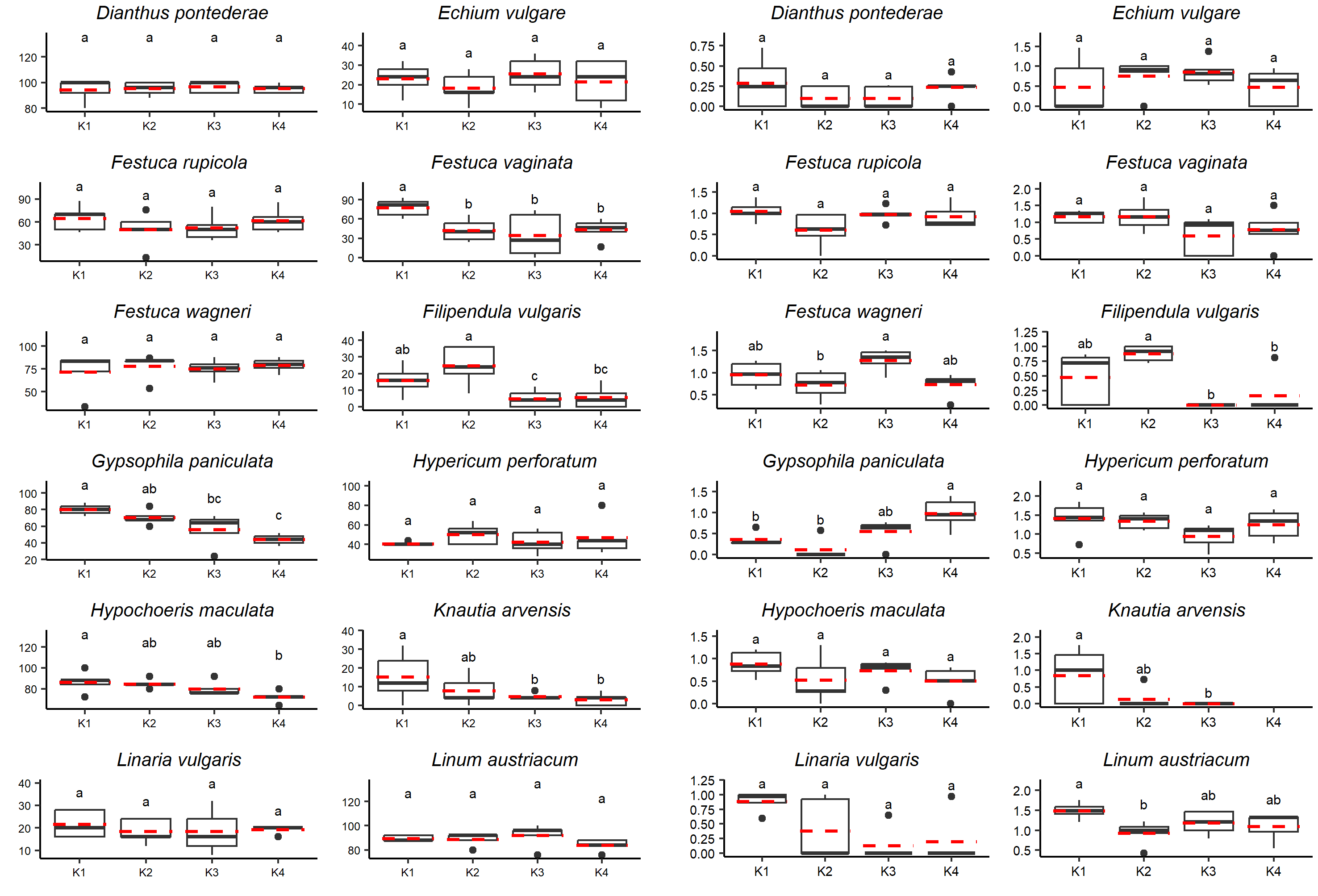

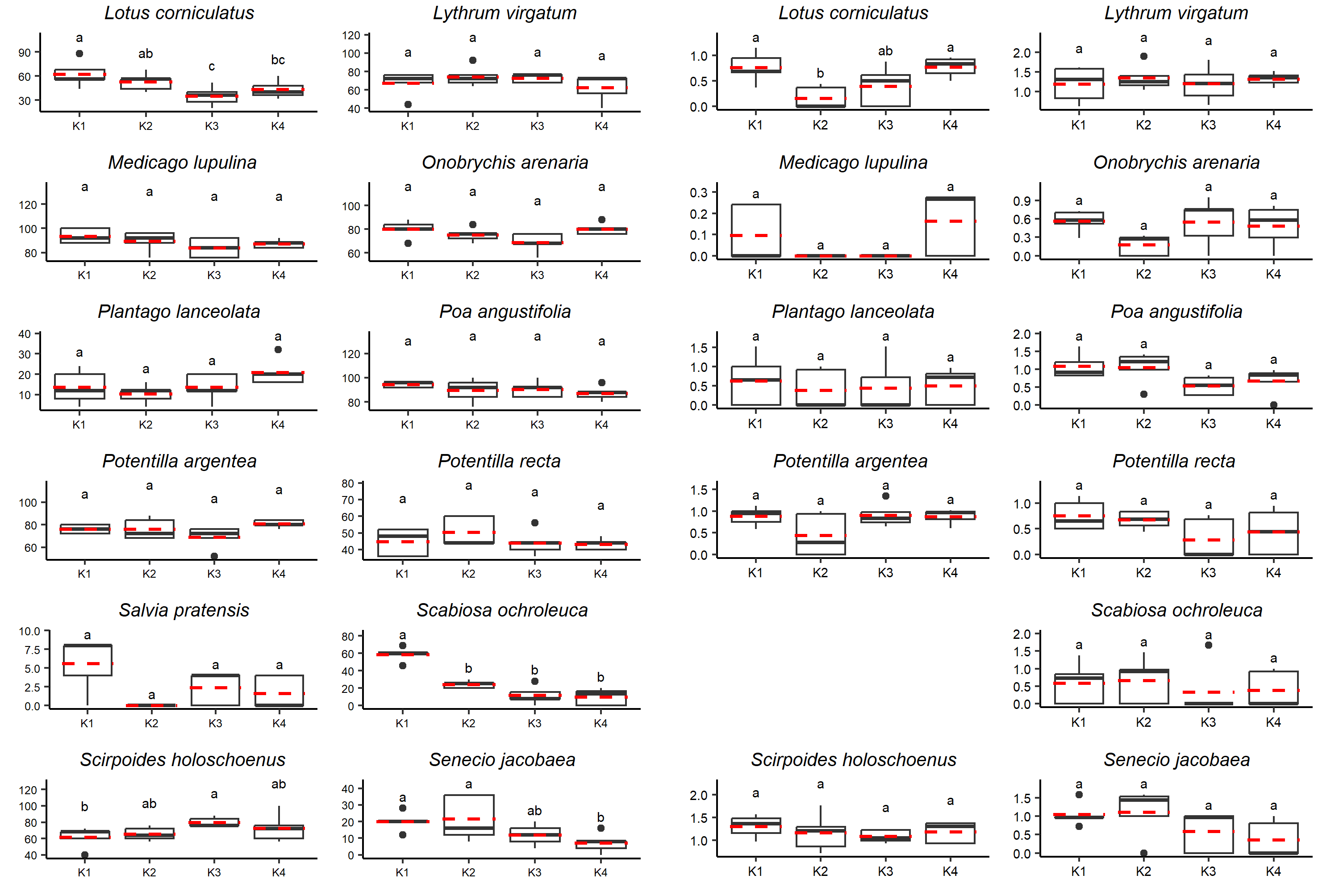

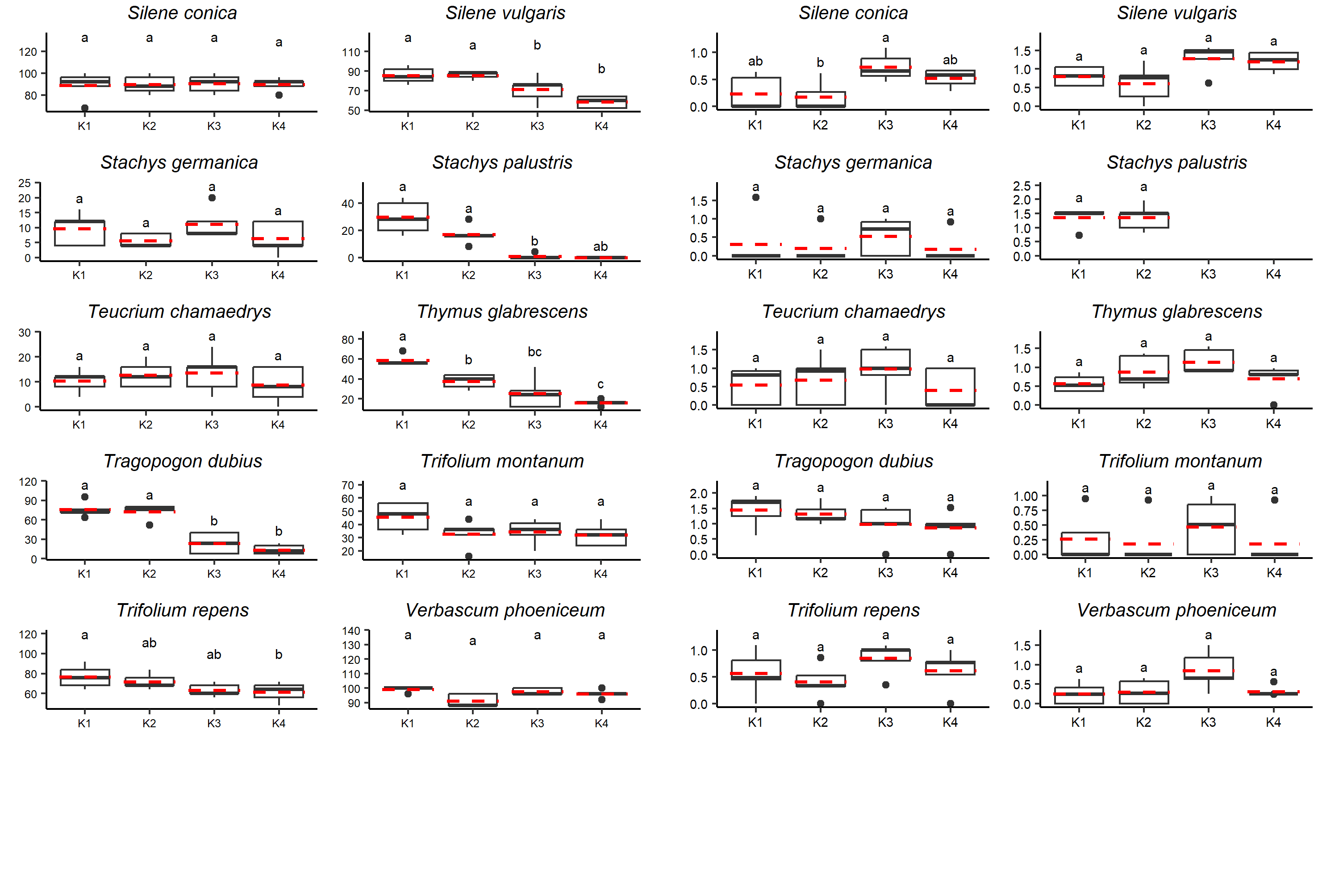

Supplement: Supplementary file 1 — Supplementary Material 1 [file 12862_2025_2424_MOESM1_ESM.docx]
